# Cannabis is associated with clinical but not endoscopic remission in ulcerative colitis: A randomized controlled trial

**DOI:** 10.1371/journal.pone.0246871

**Published:** 2021-02-11

**Authors:** Timna Naftali, Lihi Bar-Lev Schleider, Fabiana Scklerovsky Benjaminov, Fred Meir Konikoff, Shelly Tartakover Matalon, Yehuda Ringel

**Affiliations:** 1 Institute of Gastroenterology and Hepatology, Meir Medical Center, Kfar Saba, Israel; 2 Sackler School of Medicine, Tel Aviv University, Tel Aviv, Israel; 3 Research Department, Tikun-Olam – Cannbit Pharmaceuticals, Tel-Aviv, Israel; 4 Clinical Research Center, Soroka Medical Center and Faculty of Health Sciences, Ben-Gurion University of the Negev, Be’er-Sheva, Israel; Kaohsiung Medical University, TAIWAN

## Abstract

**Background:**

Cannabis is often used by patients with ulcerative colitis, but controlled studies are few. We aimed to assess the effect of cannabis in improving clinical and inflammatory outcomes in ulcerative colitis patients.

**Methods:**

In a double-blind, randomized, placebo-controlled trial, patients received either cigarettes containing 0.5 g of dried cannabis flowers with80mgTetrahydrocannabinol (THC)or placebo cigarettes for 8 weeks. Parameters of disease including Lichtiger disease activity index, C reactive protein (CRP), calprotectin, Mayo endoscopic score and quality of life (QOL) were assessed before, during and after treatment.

**Results:**

The study included 32 patients. Mean age was 30 years, 14 (43%) females. Lichtiger index improved in the cannabis group from 10.9 (IQR 9–14) to5 (IQR 1–7), (p<0.000), and in the placebo group from 11 (IQR 9–13) to 8 (IQR 7–10)(p = 0.15, p between groups 0.001). QOL improved in the cannabis group from 77±4 to 98±20 (p = 0.000) but not in the placebo group (78±3 at week 0 and 78±17 at week 8;p = 0.459; p between groups 0.007). Mayo endoscopic score changed in the cannabis group from 2.13±1 to 1.25±2 (p = 0.015) and in the placebo group from 2.15±1to 1.69±1 (p = 0.367, p between groups 0.17).

**Conclusion:**

Short term treatment with THC rich cannabis induced clinical remission and improved quality of life in patients with mild to moderately active ulcerative colitis. However, these beneficial clinical effects were not associated with significant anti-inflammatory improvement in the Mayo endoscopic score or laboratory markers for inflammation.(clinicaltrials.gov NCT01040910).

## Introduction

Ulcerative colitis (UC) is an inflammatory bowel disease (IBD) characterized by inflammation of the large intestine. The incidence of UC has increased over the past few years with a higher prevalence in the developed world [1, 2]. The disease poses a significant personal and socioeconomic burden due to its effects on patients’ quality of life, daily functioning and use of healthcare system. The overall response to currently available treatments is limited to 40–60% [3, 4], and secondary loss of response occurs in about 50% of the patients [5]. Moreover, the current treatment carries many long-term risks including malignancies, infections, and decreased bone density. Therefore, it is not surprising that many patients with IBD seek alternative treatments for their illnesses. A common such alternative treatment is the use of cannabis. Indeed, epidemiological data indicate that as many as 15% of patients with IBD use cannabis [6, 7].

Cannabinoids have been shown to decrease motility and secretions in the gastrointestinal tract [8, 9]. They also have an important role in the regulation of inflammatory response in the colon [10]. In several models of murine colitis Cannabinoids were also shown to improve inflammation [11].

However, despite the growing number of IBD patients using medical cannabis, data about its clinical therapeutic efficacy is limited. Several studies reported the prevalence of cannabis use among IBD patients and suggested clinical benefit, but they were not randomized controlled studies and did not include information about the doses, extent of endoscopic disease and the effect of the treatment on disease activity and inflammatory markers [6, 7].

We have previously conducted several studies to look at the effect of medical cannabis in patients with IBD. In an observational prospective open label study on30 patients with Crohn’s disease we found a significant clinical improvement with an average decrease in Harvey Bradshaw index from 14 ± 6.7 to 7 ± 4.7 (P < 0.001). We also found that the improvement was sustained over an average period of 2 years (ranging from 3 months to 9 years) [12]. In a double-blind placebo-controlled study of 21 patients with Crohn’s disease who were treated with cannabis over a period of 8 weeks, we found a significant improvement in Crohn’s disease activity index (CDAI) in the cannabis active group compared to the placebo group (152±109 vs. 306 ±143, P <0.05) [13]. However, the results of studies investigating the effect of cannabis in IBD are not always consistent. For example, in a study on 20 patients with Crohn’s disease who were treated with cannabidiol vs. placebo over 8 weeks, we did not find significant improvement in CDAI compared to placebo, [14]. Similarly, a recent study by Irving PM et al. [15] failed to show significant difference in remission rate in UC patients who were treated with cannabidiol(n = 29) vs. placebo (n = 31)over a period of 10 weeks. Taken together, the current data on the beneficial effect of cannabis in patients with IBD is limited due to the small number of prospective placebo-controlled studies and the focus on clinical outcome without comprehensive assessment of the effect of this treatment on objective disease parameters including mucosal inflammation and inflammatory markers. Thus, the key question of whether the reported beneficial clinical effect of cannabis in patients with IBD relates to relief of symptoms or improvement in patients’ ability to tolerate their symptoms, *or* to the anti-inflammatory effects of cannabis remained unanswered.

The aim of the current study was to investigate the clinical, laboratory and endoscopic effects of medical cannabis in patients with mild to moderate UC.

We hypothesized that the use of cannabis as an adjunct therapy in patients with mild to moderate UC will be associated with better clinical outcomes compared to placebo and that this beneficial effect of treatment will be associated with improvement in objective inflammatory disease parameters including laboratory and colonic mucosal markers for inflammation.

## Materials and methods

### Study design

We conducted a single-center, prospective, randomized, double-blind, placebo-controlled, parallel-arm clinical study. The protocol included a two-week *screening period* to evaluate for baseline symptoms, an eight-week *treatment period* and a two-week *follow-up period* after the intervention was discontinued. Non-responders were offered to participate in an open arm eight-week *treatment period*.

**Fig 1:**
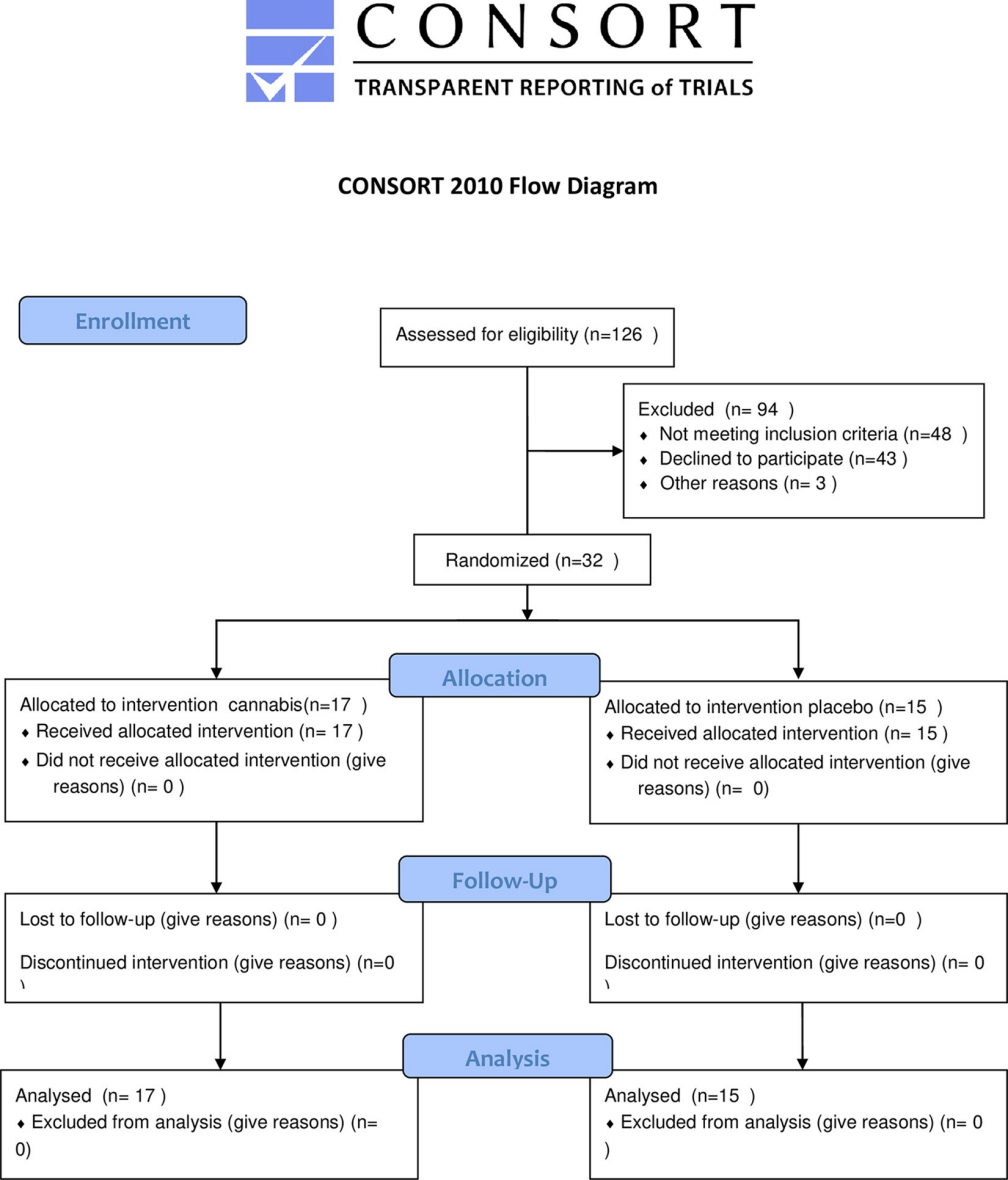
CONSORT 2010 flow diagram.

Patients were evaluated by medical interview, physical examination, blood, and stool tests at baseline (end of screening; week 0), after two weeks of study intervention (week 2), end of intervention (week 8), and end of the follow-up period (week 10). Colonoscopy was performed at screening (week 0) and after 8 weeks of treatment. (Fig 1, consort checklist).

#### Participant eligibility criteria

The study population included male and female patients age 20 to 80 years with mild to moderate UC diagnosed at least three months prior to enrollment. Mild to moderate disease severity was determined by Lichtiger Scoring Index of ≥4 and Mayo endoscopicsubscore≥1 [16]. Exclusion criteria included the use of cannabis, whether medical or recreational, pregnant or lactating, severe UC (Mayo score >10), proctitis (i.e. inflammatory segment of less than 15 cm), known psychiatric diagnosis or addiction traits based on self- reporting or noted in the patient’s electronic medical record. Patients were allowed to continue their chronic UC medications as long as they were on a stable dose; specifically, at least 4weeks for 5 ASA and at least 3 months immunomodulators and biologic treatments. Steroids were permitted if the patients were on a stable dose for at least 8 weeks prior to enrollment. Patients were specifically asked to avoid any change in their stable medications and study medication during participation in the study.

### Study compounds

Treatment was provided in the form of cigarettes. The cigarettes were machine made to ensure they were identical and comprised of dried flowers of genetically identical plants of *Cannabis sativa* var. Indica "Erez" (courtesy of Tikun-Olam Ltd., Tel Aviv, Israel). Every batch used in the study was analyzed and the content was 16% THC (80mg THC), 0.5% CBG, 0.1% CBD and traces (less than 0.1%) of CBC, CBDV and Δ8THC. Terpenes content was: Myrcene, β-caryophyllene, Selina-3,7(11)-diene, γ-Selinene, 10-epi-γ-eudesmol, β-eudesmol, guaiol, α-pinene(analysis performed in the Lumirlab, Hebrew University Biotechnology Park *Jerusalem*, *Israel*. Tel: +972 (73) 733 0300).

The placebo cigarettes contained cannabis flowers from which THC had been extracted as previously described [13]. In short, dried flowers of *Cannabis sativa* var. Indica "Erez" (Tikun-Olam Ltd., Tel Aviv, Israel), known to contain 23% THC and <0.5% CBD and other cannabinoids weres oaked in 95% ethanol for two weeks. The procedure was repeated 3 times. Following this, the flowers were covered with a mixture of herbal spirits and 0.025% *Saccharomyces cerevisiae* var. "18" (Courtesy Rimontest Ltd., Haifa, Israel) for three more days and then allowed to dry in the ambient air with ventilation for 72 hours. The final product was tested for cannabinoids and shown to possess <0.4% THC with undetectable amounts of all other cannabinoids including CBD.

### Blinding and randomization

Before the study began cannabis and placebo cigarettes were prepared by the cannabis dispensary personnel that had no access to the patients, in packages that were numbered randomly. The code was kept outside the hospital in "Tikun-Olam" and was accessible only to people who had no access to the patients. Patients were randomly assigned using a block method in blocks of 5 [17] in a 1:1 ratio to receive either medical cannabis or placebo. Patients and investigators were blind to the treatment throughout the duration of the study and the data analysis.

### Study intervention

Treatment was provided in the form of cigarettes. We chose this form because in "real life" it is reported by patients as the most effective form, with a rapid response and improvement of pain and general wellbeing. Therefore, despite the known hazards of smoking, we thought it should be the first form to be investigated [12]. Patients were required to start gradually, smoking half a cigarette (0.25gr) in the first day and increasing by 0.25 gr until a final dose of 0.5 gr twice daily was reached. To assess adherence, patients were required to bring the packages on each visit and the number of remaining cigarettes was counted.

### Outcome assessment

The primary endpoint was statistically significant improvement of the Lichtiger score, Secondary end points were: statistically significant improvement of the bowel movements, abdominal pain and quality of life. Another secondary endpoint was statistically significant improvement of the Mayo endoscopic score.

### Assessment of clinical effect

Patients were evaluated by medical interview, physical examination, blood and stool tests. Demographic data, smoking history, past medical history (including history of drug abuse and psychiatric co-morbidity), ulcerative colitis history, past and present medications, family history of IBD, results of recent blood tests, last endoscopic and imaging findings were collected from patients’ records.

For clinical assessment, we used the overall Lichtiger Score [18] as well as additional sub-analysis on Lichtiger Score specific variables of interest including the number of bowel movements per day, abdominal pain and rectal bleeding. The primary outcome was statistically significant reduction of the Lichtiger score after 8 weeks of intervention.

Quality of life (QOL) was assessed at baseline (week 0) and end of the intervention (week 8) using the Short Form (SF36)survey [19].

Patients were also asked to report their general satisfaction with the treatment on a 7 point Likert scale (1 = not at all satisfied to 7 = very satisfied) and overall improvement on specific symptoms including general health, appetite, libido and concentration on a 5 points Likert scale (1 = significant improvement to 5 = worsening).

### Assessment of effect on inflammation

Inflammatory activity was assessed with laboratory blood tests, stool calprotectin, and endoscopic parameters. Blood tests included complete blood count, liver and kidney function and C-reactive protein (CRP). Colonoscopies were performed at baseline (week 0) and end of intervention (week 8) by physicians who were blinded to the patient’s study treatment. Endoscopic disease activity was assessed using the Mayo score [20].

All side effects, including symptoms of drug addiction as defined by the DSM- IV [21] were captured at week 2 and week 8 and rated for severity on a 0 to 7 scale.

### Statistical analysis

Categorical variables were reported as number and percentage. Continuous variables were evaluated for normal distribution using histogram and QQ plot. Baseline characteristics at first visit evaluation and third visit were compared between groups using independent sample t-test or Mann-Whitney test for continuous and ordinal variables, while Chi-square test or Fisher exact test were used for categorical variables. In each group, differences between the first and third visits were tested using paired sample t-test or Wilcoxon test for continuous and ordinal variables, while McNemar test was used for categorical variables. Generalized estimating equations models were used to observe changes between the groups at two time points, the first week and the 8 weeks visits. This was evaluated using interaction between time and group.

Corrections for multiple comparisons were done using the False Discovery Rate method [22].

In order to identify a4 point difference in the Lichtiger score between the two groups after 8 weeks, we used a standard deviation of 2.5, [23] an alpha of 0.01and a power of 90%. The calculated sample size was 14 patients in each group. Taking into account the possibility of 10% dropout we aimed at 16 patients in each group.

All statistical tests were 2-sided, p<0.05 was considered statistically significant. SPSS software was used for statistical analysis (IBM SPSS statistics for windows, ver. 25, IBM Corp, Armonk, NY, USA).

#### Ethical considerations

The study was approved by the Ministry of Health cannabis authority ethics committee and the Meir Medical Center ethics committee. All participants provided informed consent before any study-related procedure was carried out. All methods were carried out in accordance with relevant guidelines and regulations. The study protocol and results are registered on the clinicaltrials.gov website. NCT01040910, first posted 30 December 2009, and modified on October 2013.

## Results

A total of 126 patients were screened, among them,43 did not consent, 39 had inactive disease with a Lichtiger score ≤1, inclusion criteria were not met by 9 patients, and3 were already taking medical cannabis treatment. Thus, 32 patients were recruited and all completed the study.

The mean age was 30, range 26–40, 14 (43%) women. Left-sided colitis was noted in 8 (25%) and extended or pancolitis in 24 (75%) patients. The mean length of the colonic involved segment was 46±20 cm. Twenty-four (75%) patients had never smoked tobacco, 6 (18%) smoked in the past and 2(6.3%) were still smoking during the study. Demographic data are presented in Table 1.

**Table 1:** Demographic data.

	Cannabis	Placebo	p value
No.	17 (53%)	15 (47%)	
Age(years)	30 (27–37)	30 (26–40)	0.882
Gender (M/F)	7/10	11/4	0.067
Duration of disease (years) (median±IQR)	8 (2–12)	6 (2–10)	0.970
Current smoking	0 (0%)	2 (13%)	0.411
IBD in family	6	6	0.7

IBD related treatments prior to enrollment included 5 (15%) patients using steroids, 5(15%)immunomodulators, and 6 (18%) biologics. Seven patients did not respond or had lost response to TNF inhibitors after at least a full induction dose (Table 2). No change in UC treatment was made during the study.

**Table 2:** Medications.

	Past			Present		
	cannabis	placebo	p value	cannabis	Placebo	p
5 ASA	17 (100%)	14 (93%)	0.615	7 (41%)	10 (66%)	0.596
Antibiotics	4 (44%)	5 (55%)	0.699	0	0	1
Steroids	9 (42%)	12 (57%)	0.54	2 (12%)	3(20%)	0.659
Immunomodulators	7 (41%)	8 (53%)	0.615	2 (12%)	3 (20%)	0.659
Biologics	7 (41%)	4 (27%)	0.615	4 (23%)	2 (13%)	0.659

Lichtiger disease activity index improved in the active arm group from 10.9 (IQR 9–14) to5 (IQR 1–7, p<0.001), and in the placebo group from 11 (IQR 9–13) to 8 (IQR 7–10, p = 0.37). (p between groups 0.006). When looking at the delta of the Lictiger score, the average change was 6.4 ±3.1 in the cannabis group and 2 ±2.5 in the placebo group (p<0.05), only two patients, both from the placebo treated group, had an increase in the Lichtiger score, but the change was less then 3 points, and thus not defined as a disease flare. The number of bowel movements per day decreased from 2.6 (IQR 2–4) to 1 (IQR 0–1, p<0.001)and from 2.6 (IQR 2–4) to 2 (IQR 2–3, p = 0.168) in the active arm and placebo groups respectively (p between groups 0.006). The number of patients who reported severity of abdominal pain of ≥ 2 decreased from 10 (59%) at baseline to 1 (6%) after 8 weeks of treatment(p = 0.006) in the cannabis group and from9 (60%) to8 (55%),(p = 0.429) in the placebo group, (p between groups = 0.04). The number of patients who reported blood in stool decreased from 13 (76%) to 5 (30%) in the cannabis group (p = 0.015). and from 9 (60%) to 6 (40%) in the placebo group (p = 0.589)(p between groups = 0.64) (Table 3).

**Table 3:** Effect of cannabis on disease activity.

	Cannabis			Placebo			p Cannabis vs placebo	
	visit 1	visit 3	p	visit 1	visit 3	p	visit 1	visit 3
Lichtiger score	10.9 (IQR 9–14)	5 (IQR 1–7)	0.001	11 (IQR 9–13)	8 (IQR 7–10)	0.37	0.914	0.006
Bowel movements (median IQR)	2.6 (IQR 2–4)	1 (IQR 0–1)	0.001	2.6 (IQR 2–4)	2 (IQR 2–3)	0.168	0.914	0.006
Abdominal pain≥2	10 (59%)	1 (6%)	0.006	9 (60%)	8 (55%)	0.429	0.914	0.04
Blood in stool	13 (76%)	5 (30%)	0.015	9 (60%)	6 (40%)	0.589	0.73	0.645
Endoscopic Mayo score	2.13±1	1.25±2	0.015	2.15±1	1.69±1	0.367	0.914	0.374
QOL	77±4	98±20	0.001	78±3	78±17	0.631	0.914	0.026
WBC	6.7±0.4	6.8±0.4	0.044	8.9±0.7	7.9±0.8	0.37	0.587	0.393
Hb	12.9±0.6	13.1±0.5	0.776	13.6±0.5	13.1±0.5	0.828	0.733	0.911
CRP	1.8±0.2	2.8±1.9	0.652	0.8±0.4	1.1±0.3	0.828	0.578	0.843
Calprotectin	170±33	134±33	0.072	226±34	218±67	0.9	0.688	0.393
Weight	68±4.7	66±5.2	0.24	60±1.8	58±2.3	0.367	0.578	0.286

QOL improved in the cannabis group from 77±4 to 98±20 (p = 0.001) but not in the placebo group (78±3 at week 0 and 78±17 at week 8;p = 0.631; p between groups 0.026) (Table 3).

Colonoscopy at baseline and at the end of treatment was performed in 29 out of 32 (90%) patients, Mayo endoscopic score improved in the cannabis-treated group from an average of 2.13±1 to 1.25±2 (p = 0.015) and in the placebo group from 2.15±1to 1.69±1 (p = 0.367). However, pre- to post-intervention differences between the groups (delta between pre intervention and post intervention score)did not reach statistical significance(1.25±2and 1.69±1 in the study and placebo groups, respectively, p = 0.374).

Baseline to end of 8 weeks treatment laboratory parameters of inflammation, including blood count, CRP, and fecal calprotectin did not change in both groups (Table 3).

When asked about the effect of treatment on specific symptoms, patients in the cannabis group reported improvement in their general health, appetite, libido, concentration, and pain. The placebo group did not report similar changes. General satisfaction with treatment was high among the cannabis treated group. Interestingly, the improvement was noted within one week (Table 4).

**Table 4:** Clinical effect of cannabis (assessed by patient questioning*).

Parameter:	Study group	Placebo group	p
General health (yes/no)	14/1 (82%)	1/14 (6.7%)	0.003
Mood	3.3±1.1	3.6 ±1.1	0.384
Memory	4.5±0.7	4.07±2.5	0.168
Appetite	2.5±1.2	3.7±0.8	0.019
Concentration	2.0±1.1	3.9±0.5	0.002
Sleep	4.47±0.7	4.07±0.4	0.178
Daily function	3.47±0.7	4±0	0.099
Alertness	3.85±0.9	3.9±0.2	0.852
Libido	1.93±0.7	4±0	0.001
Pain	2.7±1.3	3.9±0.25	0.013
Abdominal swelling	3.7±1.1	4±2.5	0.446
General satisfaction from treatment	2.4±1.5	5.6±1.6	0.001
How long did it take to feel the change (1 = Immediately, 2 = within 1 week, 3 = within 2 weeks, 4 = no change)	0.9±0.7	3.8±0.4	0.001

* On a grade from 1 to 7, 1 = improved, 4 = no change, 7 = deteriorated

The reported side effects were minor and did not lead to cessation of treatment in any patients (Table 5)

**Table 5:** Reported side effects.

	Cannabis	placebo	p
Cough	7 (41%)	3 (20%)	0.272
Dizziness	6 (35%)	1 (6%)	0.272
confusion	5 (29%)	1(6%)	0.304
Difficulty to stop use	5 (29%)	2 (12%)	0.543
Behavioral change	4 (23%)	0 (0%)	0.27
Restlessness	2 (11%)	0 (0%)	0.543
Shortness of breath	1 (6%)	0 (0%)	0.543
Decreased memory	0 (0%)	6 (40%)	0.153
Hallucinations	0 (0%)	0 (0%)	0.883

Of the 32 patients who completed the study, 17 patients continued active cannabis use and follow up in our clinic for an additional period of one year. Eight were from the cannabis study group and 9 from the placebo group. The majority (n = 14) of these patients did not need any medical intervention throughout this one-year follow-up. Two patients needed a course of steroids and one patient started treatment with Vedolizumab. At the end of this year, 11 patients underwent a colonoscopy and 10 of them had a Mayo score of 1–0, whereas before initiation of cannabis 2 had a score of 3 and 8 had a score of 2.

Reasons for not continuing follow up (n = 15 patients) included: lost contact (n = 5), change of residence (n = 3) and wish to stop cannabis (n = 7). The reasons for discontinuing cannabis treatments were intolerance (n = 4), clinical deterioration (n = 2) and planning to become pregnant (n = 1).

## Discussion

Epidemiological studies indicate that between 15–45% of patients with IBD use cannabis [6, 7] and anecdotal clinical reports suggest improvement in patient’s wellbeing and IBD-related symptoms [7, 12, 24]. In addition, preclinical animal and laboratory investigational models have demonstrated anti-inflammatory effects of cannabis, thus further supporting a potential benefit of using cannabis in patients with IBD [7, 10, 11].

The endocannabinoid system has an important role in the regulation of inflammatory response in the colon [10]. Cannabinoids were shown to ameliorate colitis in various murine models of colitis, with an anti-inflammatory effect mediated thorough activation of the cannabinoid receptors CB1 and CB2, inhibition of the endocannabinoid degrading enzymes Monoacylglycerol lipase(MAGL) and fatty acid amid hydrolase (FAAH), and activation of the G protein-coupled receptor 55 (GPR55) and Transient receptor potential vanilloid 1 (TRPV1) receptors [25, 26].

However, despite the increasing anecdotal reports suggesting a clinical benefit of cannabis in patients with IBD and the accumulating data on its intestinal, and specifically colonic anti-inflammatory effects in animal models of IBD, only a few prospective, placebo-controlled studies have been conducted. Furthermore, most of the studies focused on clinical outcomes and did not include investigation of objective anti-inflammatory effects [6, 12, 24]. Therefore, the question whether the observed effect is limited to symptomatic improvement or due to a reduction in inflammation remains open.

In the current study, we investigated clinical as well as endoscopic and laboratory responses to cannabis treatment in patients with UC in a randomized placebo-controlled study. Unlike previous studies we were specifically interested to see if the clinical effects of cannabis treatment will be associated with a reduction of inflammation.

From a clinical perspective, we found that treatment with cannabis led to a significant reduction in the Lichtiger Disease Activity Index and improvement in major IBD-related clinical symptoms including abdominal pain and number of bowel movements per day. We also observed a significant improvement in quality of life, general health, appetite, libido, concentration, and patient satisfaction with the treatment.

Regarding the effect on inflammation, we found a significant pre- to post-intervention improvement in the Mayo endoscopic score in both study groups, This effect was greater in the cannabis than in the placebo group, however it did not reach statistical significance in between groups’ analysis. In addition, we could not find significant pre- to post-intervention changes in laboratory markers of inflammation including blood count, CRP and fecal calprotectin within the cannabis and the placebo groups, nor in between groups analysis.

In a study from our group using THC rich cannabis in patients with Crohn’s disease, we found significant clinical improvement, reduction of CDAI and improved quality of life, but no change in CRP [12, 13]. Similarly, Irving et al, who gave Cannabidiol (CBD)to patients with UC showed clinical improvement in partial Mayo score without improvement in inflammatory markers including endoscopic Mayo score [15]. The lack of association between clinical beneficial observation and anti-inflammatory effects could result from differences in the effect of various chemical components of cannabis. The two major active components of cannabis are cannabidiol (CBD) and Δ9-tetrahydrocannabinol (THC). While CBD works mainly peripherally without a central effect, THC works mainly centrally and is responsible for the dominant psychoactive effects of cannabis [25]. These two components seem to act synergistically onCB1 and CB2 at the level of the enteric nervous system [26].

In the current study, we used THC rich cannabis., Thus it is quite likely that the observed effect was rather central than peripheral and therefore resulted in a weaker anti-inflammatory effect. Another possibility is that the onset of the central clinical effect is faster while the anti-inflammatory effect may take longer and therefore we could not detect an accompanying effect on peripheral inflammatory markers in this relatively short, 8 weeks study. Lastly, through its effect on CB1 and CB2 receptors in the gut, cannabis also affects GI physiology including reducing intestinal motility, increasing fluid absorption and inducing analgesia [8, 24]. Therefore, it is possible that the symptomatic improvement observed in our study reflects the effect on intestinal physiology without a significant effect on inflammation.

Smoking tobacco is known to have a positive effect in UC. We chose smoking as a mode of cannabis consumption because this is the most common form used by patients in "real life". However, this may have lead to the high rate of response in the placebo group.

Regardless of the mechanism by which cannabis exerts its clinical effect, the endpoint of patient wellbeing, quality of life and daily functioning is of no lesser value than improvement in inflammation.

Overall, cannabis was well tolerated in our study. Patients reported only minor side effects, mostly dizziness (n = 6, 35%) and confusion (n = 5,29%) and none of our patients dropped out of the study due to side effects. A study among 3,341 patients using cannabis reported the most common side effects of dry mouth (26%) and feeling foggy (23%). These side effects were associated with THC and much less with CBD [27, 28]. In the study by Irving et al [15], doses of up to 500mg/day of CBD produced a high rate of side effects which led to violation of protocol and/or dropouts by 41% of the participants. The low level of side effects and lack of drop out in our study could be explained by our treatment protocol which started cannabis treatment at a low dose and increased the dose gradually, hence enabling the patients time to develop tolerance to the treatment.

Our study has several strengths including the stable dose of cannabis used, the placebo-controlled design and the examination of inflammatory parameters, including endoscopic and laboratory markers for disease activity, in addition to clinical parameters. The weaknesses of the study are the small sample size, short duration of the study, lack of histological data and the inherent difficulty of blinding cannabis use. Future studies are needed with higher sample sizes, and combining other populations. Another weakness is the consumption of cannabis as cigarettes. Although in "real-life" most patients who report beneficial effects of cannabis consume it by smoking, this mode of delivery is not advisable and could not be acceptable for medical treatment. Other healthier modes of consumption should be investigated. Vaping could be an option since vaporizers do not produce toxic compounds formed by pyrolysis and the pharmacokinetics of vaporized and smoked cannabinoids is comparable. Oral consumption is another possibility, but oral THC formulations exhibit variable absorption and undergo extensive hepatic first-pass metabolism, producing lower peak plasma concentrations relative to inhalation. Further studies are needed to evaluate the various modes of cannabis consumption and select those that safest and most efficient [29–31].

Placebo controlled studies are particularly challenging when using psychoactive substances. We tried to overcome this difficulty by recruiting only patients who did not experience previous cannabis use. Indeed, at least 3 patients receiving placebo were convinced they were receiving cannabis, but we do not have this data on all the study participants.

Our study was designed as a short (8 weeks) intervention study. However, we had the opportunity to follow a third of the patients for another year and found that endoscopic remission was retained (with a Mayo score of 0–1) in 10/11 patients. This long-term remission suggests a possible durable beneficial effect of cannabis. Larger, long-term studies are warranted to investigate this finding.

## Conclusion

This study demonstrates that treatment with THC-rich cannabis in patients with mild to moderate UC is associated with clinical improvement. Our findings indicate that the reported cannabis-induced clinical effect is not directly linked to an anti-inflammatory effect of cannabis. However, the results demonstrate a signal for associated reduction in mucosal inflammation in patients with UC. This preliminary observation requires additional investigation in larger and longer intervention clinical studies. Such studies will enable us to determine whether cannabis has mainly a symptom relieving role or a more specific anti-inflammatory therapeutic effect. Future research should focus on alternative ways of providing cannabis (other than smoking), and explore various cannabinoid compounds in order to reveal the most effective and safe mode of cannabis use by patients with IBD.

## Supporting information

S1 Checklist (DOCX)

S1 File (DOCX)

S2 File (DOCX)
