# Supplementary material for: Cannabis is associated with clinical but not endoscopic remission in ulcerative colitis: A randomized controlled trial
Source: PLoS One. 2021 Feb 11;16(2):e0246871. doi: 10.1371/journal.pone.0246871 (PMC7877751; doi:10.1371/journal.pone.0246871)
Supplement: S1 Checklist — (DOCX) [file pone.0246871.s001.docx]

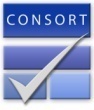
CONSORT 2010 checklist of information to include when reporting a randomised trial*

| Section/Topic | Item No | Checklist item | Reported on page No |
| --- | --- | --- | --- |
| Title and abstract | | | |
|  | 1a | Identification as a randomised trial in the title  Yes, please see title | 1 |
|  | 1b | Structured summary of trial design, methods, results, and conclusions (for specific guidance see CONSORT for abstracts)  In the abstract page 3 | 3 |
| Introduction | | | |
| Background and objectives | 2a | Scientific background and explanation of rationale  Introduction pages 4 and 5 | 4 |
|  | 2b | Specific objectives or hypotheses  Page 5, 14 lines from bottom of page | 4 |
| Methods | | | |
| Trial design | 3a | Description of trial design (such as parallel, factorial) including allocation ratio  Page 5, trial design section | 6 |
|  | 3b | Important changes to methods after trial commencement (such as eligibility criteria), with reasons  No such changes were made | none |
| Participants | 4a | Eligibility criteria for participants  Page 6, patient population, 6 lines from top of page | 6 |
|  | 4b | Settings and locations where the data were collected  Page 6, nine lines from top of the page. | 6 |
| Interventions | 5 | The interventions for each group with sufficient details to allow replication, including how and when they were actually administered  Page 6 and 7, sections "study compound" and "assessment of clinical effect" | 6 |
| Outcomes | 6a | Completely defined pre-specified primary and secondary outcome measures, including how and when they were assessed  Pages 7 and 8, sections "assessment of clinical effect", "Assessment of effect on inflammation" | 7 |
|  | 6b | Any changes to trial outcomes after the trial commenced, with reasons  No such changes were made | none |
| Sample size | 7a | How sample size was determined  Page 8, 16 lines from top of page | 8 |
|  | 7b | When applicable, explanation of any interim analyses and stopping guidelines  Not applicable | none |
| Randomisation: |  |  |  |
| Sequence generation | 8a | Method used to generate the random allocation sequence  Page 6 5 lines from the top | 6 |
|  | 8b | Type of randomisation; details of any restriction (such as blocking and block size)  Page 6 5 lines from the top | 6 |
| Allocation concealment mechanism | 9 | Mechanism used to implement the random allocation sequence (such as sequentially numbered containers), describing any steps taken to conceal the sequence until interventions were assigned  Page 7, 5 lines from the top of the page | 6 |
| Implementation | 10 | Who generated the random allocation sequence, who enrolled participants, and who assigned participants to interventions  Page 7, 6 lines from the top of the page | 6 |
| Blinding | 11a | If done, who was blinded after assignment to interventions (for example, participants, care providers, those assessing outcomes) and how  Page 7, Eight lines from top of the page | 6 |
|  | 11b | If relevant, description of the similarity of interventions  Page 7, 4 lines from top of the page | NA |
| Statistical methods | 12a | Statistical methods used to compare groups for primary and secondary outcomes  Statistical analysis section, page 8, four lines from top of the page | 7-8 |
|  | 12b | Methods for additional analyses, such as subgroup analyses and adjusted analyses  Statistical analysis section, page 8, four lines from top of the page | 7-8 |
| Results | | | |
| Participant flow (a diagram is strongly recommended) | 13a | For each group, the numbers of participants who were randomly assigned, received intended treatment, and were analysed for the primary outcome  Page 8, two bottom lines | 8 |
|  | 13b | For each group, losses and exclusions after randomization ,together with reasons  Page 8, two bottom lines | 8 |
| Recruitment | 14a | Dates defining the periods of recruitment and follow-up  Page 6, "study population" section, 4^th^ line , recruitment was from October 2013 to December 2017 | 8 |
|  | 14b | Why the trial ended or was stopped  We reached an adequate number of patients | NA |
| Baseline data | 15 | A table showing baseline demographic and clinical characteristics for each group  Page 9 table 1 | 10 |
| Numbers analysed | 16 | For each group, number of participants (denominator) included in each analysis and whether the analysis was by original assigned groups  Page 9 table 1and 2 | 8, 10 |
| Outcomes and estimation | 17a | For each primary and secondary outcome, results for each group, and the estimated effect size and its precision (such as 95% confidence interval)  Page 10 table 3 | 9,11 |
|  | 17b | For binary outcomes, presentation of both absolute and relative effect sizes is recommended | NA |
| Ancillary analyses | 18 | Results of any other analyses performed, including subgroup analyses and adjusted analyses, distinguishing pre-specified from exploratory  Page 11 | 9, 10 |
| Harms | 19 | All important harms or unintended effects in each group (for specific guidance see CONSORT for harms)  Page 12, tables 4 and 5 | 9,12 |
| Discussion | | | |
| Limitations | 20 | Trial limitations, addressing sources of potential bias, imprecision, and, if relevant, multiplicity of analyses  Page 16, 5 lines from the top of the page | 15 |
| Generalisability | 21 | Generalisability (external validity, applicability) of the trial findings  Page 16, conclusion paragraph | 13-14 |
| Interpretation | 22 | Interpretation consistent with results, balancing benefits and harms, and considering other relevant evidence  Page 16, conclusion paragraph | 13-14 |
| Other information | | |  |
| Registration | 23 | Registration number and name of trial registry  Page 3, end of abstract section | 8 |
| Protocol | 24 | Where the full trial protocol can be accessed, if available  Page 3, end of abstract section | 8 |
| Funding | 25 | Sources of funding and other support (such as supply of drugs), role of funders  Page 2, five lines from the bottom of the page | 8 |
