# Supplementary material for: Cannabis is associated with clinical but not endoscopic remission in ulcerative colitis: A randomized controlled trial
Source: PLoS One. 2021 Feb 11;16(2):e0246871. doi: 10.1371/journal.pone.0246871 (PMC7877751; doi:10.1371/journal.pone.0246871)
Supplement: S1 File — (DOCX) [file pone.0246871.s002.docx]

**Cannabis for Ulcerative colitis**

**Version 1.3**

**Submitted to the ethical committee of Meir hospital 31 March 2014**

**Approved 5 June 2014**

**Principle investigator: Dr Timna Naftali**

**Institute of gadtroenterology and Hepatology, Meir Hospital, 59 Tchernichovsky St, Kfar Saba, Israel**

**Tel: 972-97472580**

**Email:timna.naftali@clalit.org.il**

**Co-investigators:**

| **Name** | **Academic degree** | **Affiliation** |
| --- | --- | --- |
| Timna Naftali | M D | 1.Institute of Gastroenterology and Hepatology, Meir Medical Center, Kfar Saba, 4441002, Israel  2. Sackler School of Medicine, Tel Aviv University, Tel Aviv, Israel  timnanaftali@gmail.com |
| Lihi Bar-Lev Schlieder | MsC | Soroka University Medical Centre and Faculty of Health Sciences, Ben-Gurion University of the Negev lihi@tikun-olam.co.il |
| Fabiana Scklerovsky Benjaminov | M D | 1.Institute of Gastroenterology and Hepatology, Meir Medical Center, Kfar Saba, 4441002, Israel  2. Sackler School of Medicine, Tel Aviv University, Tel Aviv, Israel  fabianabe@clalit.org.il |
| Fred Meir Konikoff | Professor | 1.Institute of Gastroenterology and Hepatology, Meir Medical Center, Kfar Saba, 4441002, Israel  2. Sackler School of Medicine, Tel Aviv University, Tel Aviv, Israel  konikoff@bezeqint.net |
| Shelly TartakoverMatalon | PhD | 1.Institute of Gastroenterology and Hepatology, Meir Medical Center, Kfar Saba, 4441002, Israel  2. Sackler School of Medicine, Tel Aviv University, Tel Aviv, Israel  Matalon.Shelly@clalit.org.il |
| Yehuda Ringel | Professor | 1.Institute of Gastroenterology and Hepatology, Meir Medical Center, Kfar Saba, 4441002, Israel  2. Sackler School of Medicine, Tel Aviv University, Tel Aviv, Israel  udiringel@clalit.org.il |

**1. Rationale & background information**

Background: Inflammatory bowel disease is a relatively common disease and its incidence is increasing recently. The treatments include various immunosuppressive drugs, from ASA-5, through corticosteroids to immunomodulatory drugs and anti-TNF biological agents. However, existing treatment has multiple side effects and many patients do not respond to the variety of drugs available.

Cannabis has been used by the human race for thousands of years, and in ancient Chinese medicine, its properties have been recognized as a pain-relieving, relieving diarrhea and anti-inflammatory effect. The plant contains over 60 different components, not all of which have been isolated or identified, however, the main psychoactive effect seems to be attributed to the 9Δ teterhydrocannabinol (THC) component, while the anti-inflammatory effect is probably due to cannabidiol (CBD)

Phytocannabinoids exert their effect by activating the endocannabinoid system. This system consists of the endocannabinoids where the main ones are Anandamide, and 2-arachidonoylglycerol (2-AG), the synthesis mechanisms of these two endocannabinoids are separate from each other, but both can, once synthesized, bind to the receptors on the cell that created them, or migrate to other cells. Tissue endocannabinoid levels are strictly controlled by a mechanism of re-uptake and hydrolysis (). Two enzymes are mainly responsible for the breakdown of endocannabinoids, the first being Fatty acid amide hydrolase (FAAH) (), which is found throughout the body but at higher levels in the brain and liver. The other enzyme, monoacylglyceride lipase (MAGL), is found in nerve cell terminals.

Both endocannabinoids and plant cannabinoids act by binding to cannabinoid receptors found on many cells in the body. The two major receptors are CB1 and CB2. Both receptors belong to a family of G-protein coupled receptors. The CB1 receptor is found primarily on nerve cell terminals, both in the central and peripheral nervous systems, but also in cells of the vascular system, reproductive system, and some of the endocrine system. The CB2 receptor is predominantly found on the lymphatic system cells, with high prevalence on B lymphocytes, less on neutrophils and monocytes, and even lower on T lymphocytes.

The endocannabinoid system is also found in the digestive tract. Cannabinoid receptors were detected in the enteric nervous system, with CB1 receptors located in the myenteric, submucosal plexus, as well as at the nerve cells that innervate the longitudinal and circular smooth muscle. CB1 receptors were found not only in the gastrointestinal nerve cells but also in epithelial and smooth muscle cells. CB2 receptors are expressed on plasma cells and macrophages in the lamina propria, on epithelial cells, and on neural cells in the submucosal myenteric plexus. These receptors are upregulated in inflammatory conditions such as experimental models of inflammatory bowel disease or untreated celiac disease.

The role of the endocannabinoid system in the digestive tract is complex and involves almost every pan of this system's function. CB1 receptor agonists decrease the cholinergic tone and thus reduce gastrointestinal motility. This effect is present in both the healthy and inflamed intestine. Activation of CB1 and CB2 receptors lowers colon sensitivity to balloon-induced pain in the rat model. In the inflamed colon, CB1 and CB2 activation reduce the pain threshold. activating the CB2 receptor reduces the pain caused by bradykinin. The CB1 antagonist, Rimonabant, causing improvement of pain caused by inflammation of the colon, this finding suggests that cannabinoids play a special role in reducing pain threshold, particularly pain that is caused by inflammatory processes.

 Intestinal tissue biopsies of IBD patients found increased expression of CB1 and CB2 receptors. In several rodent inflammatory bowel disease models, endocannabinoids have been shown to reduce the severity of inflammation in the intestine. In a model of trinitrobenzene-induced sulphonic acid (TNBS) colitis mice, CB2 receptor agonists (JWH133, AM1241) were found to ameliorate colitis while antagonists (AM630) worsened it. In mice lacking the CB2 receptor (- / - CB2), this treatment had no effect.

Trials that have investigated the effect of cannabis on cytokine levels and inflammatory cell activity in humans have produced contradictory results. Several studies in which cannabis smoking for a period of several weeks in AIDS and MS patients showed no difference in the levels of the cytokines TNF-α, IL-10, IL-12p40, and IL-12p70, nor was there a difference in T cell or plasma cell proliferation. On the other hand, in long-term cannabis users, it was found that NK cell levels were lower and lymphocyte response to mitogens weaker. At the same time, there was also a decrease in IL-2 levels and an increase in IL-10 levels, ie shift of the immune response from a TH -1 reaction to a TH-2 reaction.

Prior to the design of this study, 10 Crohn's disease patients taking medical cannabis were interviewed. All patients stated that the use of medical cannabis resulted in a significant improvement in disease symptoms and a significant improvement in quality of life. Evaluation of the disease activity index (CDAI) before and after taking cannabis showed a decrease in activity from an average of 348 to an average of 108, all patients required steroids before cannabis use and all were steroid free during its use. 3 patients underwent 5 surgeries prior to cannabis use, and not one patient required surgery or hospitalization during cannabis use. Mean time to use was 3.8 years (range 3 months to 9 years). No significant side effects were observed. The findings were presented at the Israeli Association of Gastroenterology and Liver Disease held in Eilat in June 2009.

The side effects of medical marijuana use are few and minor. A review published in Canada summarized 23 studies in which marijuana was administered to patients with various diseases in a controlled and under-surveillance manner. Twenty-three controlled studies and eight observational studies were reviewed, which included data from 1932 patients treated with medical cannabis. It was found that 96% of the reported side effects were mild, the most common side effect reported by 15% of respondents (714/4779) was dizziness. More severe side effects were observed in the same prevalence in patients receiving cannabis and those receiving placebo. However, study participation time was at most 12 months and we do not have data on the effect of cannabis use for a longer time. The studies included only treatment with different oral cannabis preparations, and none included smoked cannabis. It should be noted that when adverse effects of cannabis are assessed in recreational users, they include a population of people who consume cannabis but also tend to smoke tobacco, drink alcohol and consume other drugs. Therefore it is difficult to isolate cannabis effects from other effects.

All of the above indicates that the endocannabinoid system participates in the creation and existence of inflammatory processes and therefore there is a great deal of logic in trying to harness the system to treat these conditions. There are many anecdotal reports of improvement of symptoms of inflammatory bowel disease after smoking marijuana and the impression is that the substance has a beneficial effect on the inflammatory process and that there are very few side effects, however, no placebo-controlled studies have been conducted yet.

References:

1. Fernando Rodriguez de Fonseca, Ignacio Del Arco ,Francisco Javier Bermudez-Silva, Ainhoa Bilbao,AndreaCippitelli MiguelNavaro. The Endocannabinoid System: Physiologyand Pharmacology. Alcohol & Alcoholism 2005 . **40**, 2–14.
2. Piomelli, D. The molecular logic of endocannabinoid signalling. Nature Reviews Neuroscience **2003 4**, 873–884.
3. Beltramo, M., Stella, N., Calignano, A., Lin, S. Y., Makriyannis, A. andPiomelli, D. Functional role of high-affinity anandamidetransport, as revealed by selective inhibition. Science 1997**277**,1094–1097.
4. Cravatt, B. F., Giang, D. K., Mayfield, S. P., Boger, D. L., Lerner, R. A.and Gilula, N. B. Molecular characterization of an enzymethat degrades neuromodulatoryfatty-acid amides. Nature 1996 **384**,83–87.
5. Dinh, T. P., Carpenter, D., Leslie, F. M., Freund, T. F., Katona, I.,Sensi, S. L., athuria, S. and Piomelli, D. Brainmonoglyceride lipase participating in endocannabinoid inactivation.Proceedings of the National Academy of Sciences of the United States of America 2002 **99**, 10819–10824.
6. Devane, W. A., Dysarz, F. A. 3rd, Johnson, M., Melvin, L. S. and Howlett, A. C. Determination and characterization of a cannabinoid receptor in rat brain. Molecular Pharmacology 1988 **34**, 605–613.
7. Howlett, A. C., Bidaut-Russell, M., Devane, W. A., Melvin, L. S., Johnson, M. R. and Herkenham, M. The cannabinoid receptor: biochemical, anatomical and behavioral characterization. Trends in Neurosciences 1990 **13**, 420–423.
8. Galiègue, S., Mary, S., Marchand, J., Dussossoy, D., Carrière, D., Carayon, P., Bouaboula, M., Shire, D., Le Fur, G. and Casellas, PExpression of central and peripheral cannabinoid receptors in human immune tissues and leukocyte subpopulations. European Journal of Biochemistry 1995 **232**, 54–61.
9. Duncan M, Davison JS, Sharkey KA. Endocannabinoids and their receptors in the enteric nervous system. Aliment PharmacolTher 2005;**22**:667–83.
10. Wright K, Rooney N, Feeney M, et al. Differential expression of cannabinoid receptors in the human colon: cannabinoids promote epithelial wound healing. Gastroenterology 2005;**129**:437–53.
11. Wright KL, Duncan M, Sharkey KA. Cannabinoid CB(2) receptors in thegastrointestinal tract: a regulatory system in states ofinflammation. Br J Pharmacol 2008;153:263–70.
12. A AIzzo and M Camilleri Emerging role of cannabinoids in gastrointestinal and liver diseases: basic and clinical aspects *Gut* 2008;57;1140-1155;
13. Esfandyari T, Camilleri M, Busciglio I, et al. Effects of acannabinoid receptor agonist on colonic motor and sensoryfunctions in humans: a randomized, placebo-controlled study. .Am J PhysiolGastrointest Liver Physiol 2007;293:G137–45.
14. Mathison R, Ho W, Pittman QJ, et al. Effects of cannabinoidreceptor-2 activation on accelerated gastrointestinal transit inlipopolysaccharide-treated rats. Br J Pharmacol 2004;142:1247–54
15. Izzo AA, Capasso F, Costagliola A, Bisogno T, Marsicano G, LigrestiA, Matias I, Capasso R, Pinto L, Borrelli F, Cecio A, Lutz B, MascoloN, DiMarzo V. An endogenous cannabinoid tone attenuates choleratoxin-induced fluid accumulation in mice. Gastroenterology2003;125:765–774.
16. Sanson M, Bueno L, Fioramonti J. Involvement of cannabinoidreceptors in inflammatory hypersensitivity to colonic distension inrats. NeurogastroenterolMotil 2006;18:949–56.
17. Hillsley K, McCaul C, Aerssens J, et al. Activation of thecannabinoids 2 (CB2) receptor inhibits murine mesenteric afferentnerve activity. NeurogastroenterolMotil 19:769–77.
18. Massa F, Marsicano G, Hermann H, et al. The endogenouscannabinoid system protects against colonic inflammation. J ClinInvest 2004;113:1202–9.
19. Kimball ES, Schneider CR, Wallace NH, et al. Agonists ofcannabinoid receptor 1 and 2 inhibit experimental colitis inducedby oil of mustard and by dextran sulfate sodium. Am J PhysiolGastrointest Liver Physiol 2006;291:G364–71.
20. Martin A. Storr, Catherine M. Keenan, Hong Zhang, Kamala D Patel, Alexandros Makriyannis, and Keith A. Sharkey, Activation of the Cannabinoid 2 Receptor (CB2) Protects Against Experimental Colitis Inflamm Bowel Dis 2009.
21. Dai Lu, V. Kiran Vemuri, Richard I. Duclos, Jr. and Alexandros Makriyannis The Cannabinergic System as a Target for Anti-inflammatory Therapies. Current Topics in Medicinal Chemistry, 2006, Vol. 6, No. 13
22. Wang T, Collet JP, Shapiro S, et al. Adverse effects of medical cannabinoids: a systematic review. CMAJ 2008;178:1669-78.
23. Degenhardt L, Hall W D, The adverse effects of cannabinoids: implications for use of medical marijuana CMAJ 2008; 178:1685-1686
24. Naftali, T., Bar-Lev, L., Dotan, I., et al. Cannabis Induces a Clinical Response in Patients with Crohn’s Disease: a Prospective Placebo-Controlled Study. *Clinical Gastroenterology and Hepatology* ,2013:11(10), 1276–1280.

**2. Study goals and objectives**

The purpose of this study is to investigate the effectiveness of cannabis smoking in ulcerative colitis patients compared to placebo

**2.1 Primary objectives:**

Safety

- Assessment of the safety and tolerability of smoking cigarettes containing 0.5 g of dried cannabis flowers with 80-115mg Tetrahydrocannabinol (THC) or placebo cigarettes for 8 weeks

Efficacy

- Reduction of disease activity as measured by the Lichtiger Score by at least 3 points in the study group, with a statistically significant difference in the reduction between study and placebo groups
- Reduction of at least 2 points in Mayo endoscopic score

**2.3 Secondary objectives:**

**Clinical end points**:

- Statistically significant improvement in the number of bowel movements, abdominal pain and rectal bleeding per day.
- Statistically significant improvement in Quality of life (QOL) which will be assessed at baseline (week 0) and end of the intervention (week 8) using the Short Form (SF36) survey
- Percentage of patients whose UCAI score did not increase more than 2 points
- Percentage of patients with steroid-free remission (i.e. no being administered steroid and UCAI < 2)
- Patients reported outcome of general satisfaction with the treatment on a 7 point Likert scale (1=not at all satisfied to 7 =very satisfied)

**Laboratory markers of inflammation**

- Statistically significant improvement in CRP and stool calprotectin, measured on week 0 and week 8.

**Endoscopic end points:**

- Statistically significant reduction of at the Mayo endoscopic score before and at the end of the study
- Statistically significant difference in the Mayo endoscopic score between the study and placebo groups at the end of the study

**3 Study Design:**

Single-center, to be performed in the institute of gastroenterology, Meir general hospital

Prospective, patients will be followed from screening, through week 8 (end of intervention) to week 10 (end of study), follow up will continue for patients who wish to continue treatment in Meir hospital.

Randomized, study medication will be prepared in Tikun olam, packages of cigarettes for all the study period will be made for both active compound and placebo and will be randomly numbered.

Double-blind. The code of the numbered packages, whether containing active cannabis or placebo, will be kept in Tikun Olam dispensary, outside the hospital. The people in the dispensary who prepare the packages will have no contact with the patients.

Placebo-controlled, cigarettes will contain either active cannabis dried flowers of genetically identical plants of *Cannabis sativa* var. Indica "Erez", or the same flowers after all active cannabinoids have been removed from them (as in our Crohns disease study, ref 24) All cigarettes will be machine made so they look identical.

Parallel-arm: patients recruited to the study will be randomly assigned to either active cannabis or placebo, both arms will continue in parallel.

Expected duration of the study: 3 years.

**3.1 Material and methods**:

Patient population: The study will include a total of 30 ulcerative colitis patients

**Inclusion**:

1. Patients with biopsy confirmed, active UC
2. Age ≥18 years
3. Ulcerative colitis patients, for whom the disease has been present for more than 3 months, and has been proven in conventional examinations - endoscopy or X-ray imaging. In addition, 10 more patients will be included with POUCHITIS (patients after a complete colon resection and a small bowel loop connection(
4. Patients with an active stage of the disease who are unresponsive to treatment with ASA 5 or corticosteroids or immunomodulators, or biological drugs will be recruited.
5. Patients treated by steroids will be allegeable either if they did not respond, or have become steroid-dependent, or are unable to take these drugs due to side effects or allergic reaction
6. Age of study participants 20 years or more.
7. The degree of disease activity will be determined by mayo score above 3 and Lichtiger score above 3.
8. Female subjects who are postmenopausal (absence of menses for ≥ 2 years confirmed by a follicle stimulating hormone test), or who are surgically sterilized may be enrolled. Similarly, women of childbearing potential who had a negative pregnancy test at screening, who are willing to use two medically acceptable methods of contraception for the duration of the study as well as for at least three months after cessation of cannabis treatment and who are willing to undergo pregnancy testing according to the study protocol may be enrolled.
9. Female subjects who are not breast-feeding and who have no intention to breast-feed during the term of the trial and for at least three months after cessation of cannabis treatment may be enrolled.
10. Subject able to provide written informed consent
11. Ministry of health license for medical cannabis was obtained

 Exclusion:

1. Patients with known mental disorders,
2. Patients who, in the opinion of the attending physician, have a high risk of developing an addiction or improper use of the preparation
3. Patient with a record of addiction or drug abuse
4. Patients who are recreational cannabis users
5. Patients with a known sensitivity to the preparation,
6. Pregnant women or those planning a pregnancy.
7. Patients unable to give informed consent,
8. Patients who appear to need surgery in the near future due to their illness.
9. Patients who receiving steroids at a non stable dose for less than 2 months
10. Patients receiving immunomodulators for less than 3 months, or at a changing dose.
11. Patients who started biologic treatments less than 3 months ago.
12. Patients with proctitis, i.e. the involved colonic segment is less than 15 cm.

**3.2 Intervention:**

Patients will be randomized in a 1:1 ratio into 2 groups:

1. A group that will receive cigarettes prepared from the cannabis plant for smoking twice a day. Each cigarette will contain 0.5 g of dried cannabis flowers equivalent to 8-115mg THC. The cigarettes will be comprised of dried flowers of genetically identical plants of *Cannabis sativa* var. Indica "Erez" (courtesy of Tikun Olam Ltd., Tel Aviv, Israel). Every batch used in the study will be analyzed for cannabinoid content, "Erez" is expected to contain 23% THC and <0.5% Cannabdiol (CBD), but there may be variations due to changes in the plants.
2. A group that will receive similar cigarettes from which the active substance (THC) was extracted (for details, see ref 24).

Patients will receive consecutive numbers as they enter the study. Each number will relate to a package of cigarettes prepared for the study, the numbers will be randomly assigned to the packages so that the investigators will not be able to know which is active cannabis and which is placebo.

**3.3 Study procedure**:

3.3.1 The duration of the study will be 8 weeks of active treatment, followed by 2 weeks of "washout" period.

There will be 4 study visits: screening, visit 1 at week 0, visit 2 – two weeks after initiation of treatment, visit 3 – 8 weeks after initiation of treatment, and visit 4 – 10 weeks after initiation of treatment, which will also be 2 weeks after cessation of treatment. (see table 1 for procedures done ant each visit)

Patients will undergo an endoscopic examination at the beginning and end of the study.

## 3.3.2 Blood sample collection

Blood will be collected as part of the routine medical care and analyzed in the hospitals’ laboratories. The following analyses will be performed according to the Schedule of Events

Blood tests will be taken at the beginning and end of the study to assess the activity and severity of the disease.. For cytokine and endocannabinoid future assessment, 10 mL of peripheral blood will be collected, centrifuged and serum aliquoted into 0.5 mL cryotubes and frozen at -80°C.

*Laboratory assessments:*

CBC with differential, electrolytes, blood urea nitrogen (BUN), creatinine, liver function tests [Alanine transaminase (ALT); Aspartate transaminase (AST); Alkaline phosphatase (ALP); Gamma-glutamyltransferase (GGT); L-lactate dehydrogenase (LD); Albumin and total protein], bilirubin, C-reactive protein (CRP), and fecal calprotectin.

All laboratory results will be kept in each subject’s study file.

**3.3.4 List of study visits:**

SCR visit: Weeks -2 to 0, Screening:

In order to enroll the subject into the study, the following information will be collected and procedures will be performed:

Inclusion/exclusion determination

Informed consent form signed and countersigned,

Request for license submitted to MOH

Demographics, Medical history ,Prior and ConMeds

Physical examination

Vital signs will include temperature, peripheral arterial blood pressure, heart rate, and respiratory rate. They will be obtained in a sitting or supine position after the subject has rested for 5 minutes.

Lab Assessments including: CBC with differential, electrolytes, BUN, creatinine, liver function tests [ALT; AST; ALP; GGT; LD; Albumin and total protein], bilirubin, CRP, fecal calprotectin.

Serum sampling for cytokine analysis and endocannabinoid blood levels

Lichtiger score and full Mayo score

QoL evaluation

Colonoscopy: assessment of severity of colonic inflammation will be performed during the screening period. In patients older than 50 years or with a disease duration of more than 8 years a full colonoscopy is required, including chromoendoscopy or NBI to rule out dysplasia. These patients can have a sigmoidoscopy if a full colonoscopy was performed in the last 12 months. Patients who are younger than 50 and have a disease duration of less than 8 years can have a colonoscopy to the end of the involved colonic segment, i.e. to point in the colon where inflammation is no longer seen.

Biopsies will be obtained during colonoscopy to asses severity of inflammation, rule out CMV and C diff, rule out dysplasia in the appropriate cases, and for future analysis of CB receptors and endocannabinoid system in future studies (this is clearly explained in the informed consent)

Visit 1: Week 0, Baseline assessments, start cannabis/placebo treatment:

Prior and ConMeds

Physical examination

Vital signs

Dispense placebo/cannabis

Disease activity index (Lichtiger and partial Mayo score)

QoL assessment (via Short Quality of Life Questionnaire for Inflammatory Bowel Disease (10 items), VAS, , and Tikun Olam questionnaire for cannabis and UC)

AEs/SAEs

Visit 2: Week 2, Checkup, UCAI, AEs/SAEs dispense study treatment

Prior and ConMeds

Physical examination

Vital signs

Disease activity index (Lichtiger and partial Mayo score)

AEs/SAEs

Dispense placebo/cannabis treatment

Visit 3: Week 8, Checkup, UCAI, QoL, AEs/SAEs, colonoscopy

Prior and ConMeds

Physical examination

Vital signs

Disease activity index (Lichtiger and full Mayo score)

QoL assessment

Addiction assessment questionnaire

AEs/SAEs

Lab Assessments (as in screening visit)

Colonoscopy (may be performed to the end of the involved colonic segment, i.e. to the point in the colon where inflammation is no longer seen.)

Visit 4: Week 10, Checkup, , UCAI, AEs/SAEs

Prior and ConMeds

Physical examination

Vital signs

Disease activity index (Lichtiger and partial Mayo score)

AEs/SAEs

If the patients chooses to continue being treated in Meir hospital, an appointment for the next visit will be made at a reasonable time according to patient's condition. If not, the patients will receive a summary of the medical files including description and results of all study procedures to take to his/her treating physician.

**3.4 Safety measures:**

Patients will be given telephone numbers of the institute of gastroenterology, of a study coordinator and of a study investigator, so that they have immediate contact in case they have any concerns.

Participants will be asked to complete a side effect questionnaire and an addiction assessment questionnaire at week 8.

Patients who develop a worsening condition during the study: an increase in UCAI by more than 2 points will be withdrawn from the study

Adverse Events (AEs) and Serious Adverse Events (SAEs) will be continuously assessed throughout the study, without reference to the level of possible or probable relatedness, from the beginning of cannabis/placebo administration until two weeks after cessation of cannabis/placebo

**Stopping rule:**

A patient will be considered for removal from the study, based on physician assessment, if one or more of the following occurs:

- DAI increases > 3 points
- Rescue therapy needed to control severe disease flare
- Operation connected to UC is required
- Patient withdrew consent.
- A Serious Adverse Events (SAEs) has occurred

**Breaking the Code**: if the investigator who is following the patient will deem breaking the code important for patient safety or future treatment they will consult the principle investigator, if they both agree that breaking of the code is needed they will contact Tikun Olam and the code of the specific patient will be opened. Study investigators will still not be exposed to the codes of any other patients.

The study drug, as well as the placebo, will be provided by the Tikun Olam Association, A cannabis dispensary acting with the authorization of the Ministry of Health. The study was coordinated and approved by both the Ministry of health and Dr. Yehuda Baruch, the authorized physician of the Ministry of Health to provide medical cannabis. In addition, the study will be submitted to the local ethical committee of Meir hospital. All patients will sign informed consent.

1. **Statistical analysis**

Assuming a reduction of 4 points in the Lichtiger score, for an alpha of 0.05 and a power of 80%, the calculated sample size was 12 patients in each group. However since this is a very small number we decided to increase the number of participants to 30.

All measured variables and derived parameters will be listed individually and, if appropriate, tabulated by descriptive statistics. For continuous variables summary tables will be provided giving sample size, arithmetic mean, standard deviation, median, minimum and maximum and 95% Confidence Intervals (CI). For categorical variables, summary tables will be provided giving sample size, absolute and relative frequency and 95% CI for proportions (if appropriate).

All statistical tests will be 2-sided, p<0.05 will be considered statistically significant. SPSS software will be used for statistical analysis (IBM SPSS statistics for windows, IBM Corp, Armonk, NY, USA,)

*Primary Efficacy analysis*

Efficacy outcomes will be summarized in appropriate tables by time point. Changes from baseline will be analyzed using the Signed-rank test.

Other secondary and exploratory endpoints will be summarized in appropriate tables by time-point (as available).

## Quality Assurance Program

This clinical trial may be audited according to the Clalit quality Assurance (QA) program and/or its CRO acting on behalf of Clalit medical services.

The purpose of these audits is to determine whether the study is being conducted and monitored in compliance with the protocol and recognized GCP guidelines and local regulations. These audits will also increase the likelihood that the study data and all other study documentation can withstand a subsequent inspection by any regulatory authority. Such audits, if necessary, will be pre-arranged with the site and conducted within a reasonable time frame.

## Regulatory Inspections

The study may be inspected by regulatory agencies. These inspections may take place at any time during or after the study and are based on national regulations, and ICH guidelines.

#### Budget

This study is not supported by any grant. Investigators will participate in the study as part of their work in the Gastroenterology institute of Meir hospital. Study procedures will be covered by the hospital as part of patients follow up. Study monitor will be the study monitor of the Gastroenterology institute of Meir hospital.

#### Other support for the project

The cannabis and placebo used for the project will be supplied by Tikun Olam , an authorized cannabis dispensary, who will also carry the randomization and blinding process and keep the blinding code.

#### Collaboration with other scientists or research institutions

Endocannabinoid analysis will be done with collaboration of the laboratory of Prof Yossi Tam from the school of pharmacology, Hebrew university in Jerusalem.

The course of tests and visits during the study is detailed in the table below:

| **VISIT IV** | **VISIT III** | **VISIT II** | **VISIT I** | **Screening** | **VISIT** |
| --- | --- | --- | --- | --- | --- |
| **10** | **8** | **2** | **0** | **-2** | **WEEK** |
|  |  |  |  | V | Informed consent |
|  |  |  |  | V | Request license from MOH |
| V | V | V | V | V | Checkup (including prior and conMeds, physical examination, vital signs) |
| V | V | V | V | V | lab assessments chemistry, CBC, CRP |
|  | V |  | V |  | Blood Samples for cytokine and endocannabinoid future tests |
|  | V |  | V |  | Stool calprotectin |
| V | V | V | V |  | Lichtiger score, Partial Mayo score |
|  | V |  | V |  | QOL |
|  | V | V |  |  | Side effects assessment |
|  | V |  |  |  | Addiction assessment questionnaire |
|  | V |  | V |  | Endoscopy  Full Mayo score, biopsy |
|  |  | V | V |  | Dispensation of study drug |

Study design:

Screening Visit 1 Visit 2 visit 3 visit 4

Week -2 0 2 8 10

**CRF**

**טופס מעקב אחרי משתתפים במחקר: טיפול בקנביס לקוליטיס כיבית**

**תאריך: ______ רופא מפנה: _________**

**בקור I:**

| **שם:** |  | **משפחה:** |  |
| --- | --- | --- | --- |
| **ת.ז.:** |  | **שנת לידה:** | **מין : ז/נ** |
|  |  | **טלפון:** |  |
| **מחלות רקע:** |  |  |  |
| **תרופות קבועות:** |  |  |  |
| **עישון: מעולם לא** | **בעבר, תאריך הפסקה** |  | **בהווה:** |
| **IBD במשפחה** | **כן/לא** | **איזו קרבה:** |  |
|  |  | **איזו מחלה:** |  |
| **היסטוריה של IBD:** |  | **UC** |  |
| **תאריך אבחנה I:** |  |  |  |
| **אובחן עי:** | **אנדוסקופיה** | **צ מעי דק** | **CT** |
|  | **ביופסיה** | **אחר:** |  |
| **ניתוחים: כן/לא** | **תאריך:** | **איזה ניתוח:** |  |
| **טיפול תרופתי בעבר:** | **(לפני יותר מ 1 חודש)** | |  |
| **ASA-5** | **בן/לא** | **סטרואידים** | **כן/לא** |
| **אנטיביוטיקה** | **כן/לא** | **אימורן/6MP** | **כן/לא** |
| **נוגדי TNF/** | **כן/לא**  **תאריך ערוי אחרון:** | **מטוטרקסט** | **כן/לא** |
| **טיפול ביולוגי אחר:** |  | **תאריך ערוי אחרון:** |  |
| **קטע מעי מעורב:** | **פרוקטיטיס/ מעי גס שמאלי בלבד / מעי גס שמאלי +רוחבי/ כל המעי הגס** | | |
| **מחלה נוכחית:** | **מולא /UCAI** |  |  |
| **אנדוסקופיה:** MAYO SCORE | |  | |
| **דירוג חומרת התמונה האנדוסקופית:** | |  | |
| **נלקחו ביופסיות:** | **מעי גס כן/לא** |  |  |
| **טיפול תרופתי נוכחי:**  **(חודש אחרון)** | **אנטיביוטיקה: כן/לא** | **סוג אנטיביוטיקה:** |  |
|  |  | **תאריך התחלה** |  |
|  |  | **תאריך סיום:** |  |
|  | **טיפול מקומי: פרט** |  |  |
|  | **ASA-5 כן/לא** | **תאריך התחלה** |  |
|  |  | **תאריך סיום:** |  |
|  | **סטרואידים כן/לא** | **תאריך התחלה** |  |
|  |  | **תאריך סיום:** |  |
|  | **אימורן/6MP** | **תאריך התחלה** |  |
|  |  | **תאריך סיום:** |  |
|  | **נוגדי TNF** | **תאריך התחלה** |  |
|  |  | **תאריך סיום:** |  |
|  | **ביולוגי אחר** | **תאריך התחלה** |  |
|  |  | **תאריך סיום:** |  |
| **מילא UCAI** |  | **בדיקות דם** |  |
| **מילא שאלון איכות חיים** | | **אנדוסקופיה** |  |
|  |  |  |  |

**בקור II (שבוע 2) תאריך: ________**

| אנמנזה ובדיקה גופנית |  | | | | | |
| --- | --- | --- | --- | --- | --- | --- |
| תרופות שנוטל: | תרופה | מינון | דרך מתן | אינדיקציה | התחלה | סיום |
|  |  |  |  |  |  |  |
| שינוי בטיפול התרופתי: |  |  |  |  |  |  |
| בדיקות דם: | ספירה כימיה CRP ושקיעת דם | | | | | |
| הערכת פעילות המחלה/UCAI |  | | | | | |
| מתן תרופת המחקר |  | | | | | |
| מועד לביקור הבא: |  | | | | | |

**בקור III (שבוע 8) תאריך: ________**

| אנמנזה ובדיקה גופנית |  | | | | | |
| --- | --- | --- | --- | --- | --- | --- |
| תרופות שנוטל: | תרופה | מינון | דרך מתן | אינדיקציה | התחלה | סיום |
|  |  |  |  |  |  |  |
| שינוי בטיפול התרופתי: |  |  |  |  |  |  |
| בדיקות דם: | ספירה כימיה CRP ושקיעת דם, | | | | | |
| בדיקת ציטוקינים |  | | | | | |
| הערכת פעילות המחלה  /UCAI |  | | | | | |
| אנדוסקופיה | MAYO SCORE | | | | | |
| דירוג חומרת התמונה האנדוסקופית |  | | | | | |
| מיקום לקיחת הביופסיות |  | | | | | |
| מועד לביקור הבא: |  | | | | | |

**בקור IV (שבוע 10) תאריך: ________**

| אנמנזה ובדיקה גופנית |  | | | | | |
| --- | --- | --- | --- | --- | --- | --- |
| תרופות שנוטל: | תרופה | מינון | דרך מתן | אינדיקציה | התחלה | סיום |
|  |  |  |  |  |  |  |
| שינוי בטיפול התרופתי: |  |  |  |  |  |  |
| בדיקות דם: | ספירה כימיה CRP ושקיעת דם | | | | | |
| הערכת פעילות המחלה  I/UCAI |  | | | | | |

Disease activity index for UC (Lichtiger score)

| Symptom | **Details** | **Points** |
| --- | --- | --- |
| No of bowel movements | 0-2 | 0 |
|  | 3-4 | 1 |
|  | 6-5 | 2 |
|  | 9-7 | 3 |
|  | 10 | 4 |
| Nocturnal diarrhea | No | 0 |
|  | yes | 1 |
| Blood in stools (% of Time) | None | 0 |
|  | Less than 50% | 1 |
|  | More than 50% | 2 |
|  | 100% | 3 |
| Incontinence | no | 0 |
|  | yes | 1 |
| Abdominal pain | none | 0 |
|  | mild | 1 |
|  | moderate | 2 |
|  | severe | 3 |
| General well being | Perfect | 0 |
|  | Very good | 1 |
|  | Good | 2 |
|  | Average | 3 |
|  | Poor | 4 |
|  | terrible | 5 |
| Abdominal tenderness | None | 0 |
|  | Mild and localized | 1 |
|  | Moderate and diffuse | 2 |
|  | Severe or rebound | 3 |

**Ref:** N Engl J Med 1994; 330:1841-1845**Mayo score**

| Scoring:  **Stool Frequency** 0 = Normal 1 = 1-2 stools/day more than normal 2 = 3-4 stools/day more than normal 3 = 5 or more stools/day than normal | Points |  |
| --- | --- | --- |
| **Rectal bleeding** 0 = None 1 = Visible blood with stool less than half the time 2 = Visible blood with stool half of the time or more 3 = Passing blood alone |  |  |
| **Mucosal appearance at endoscopy** 0 = Normal or inactive disease 1 = Mild disease (erythema, decreased vascular pattern, mild friability 2 = Moderate disease (marked erythema, absent vascular pattern, friability, erosions) 3 = Severe disease (spontaneous bleeding, ulceration) |  |  |
| **Physician rating of disease activity** 0 = Normal 1 = Mild 2 = Moderate 3 = Severe |  |  |
| **Assessment based on Scoring**: **Full Mayo Index Score**: ----------------------------------------- Maximum score: 12.  Higher scores indicate more severe disease (Ulcerative colitis).  A critical component of this score are the endoscopic findings.  Patient's with lower scores but with an endoscopic score of 2 or greater are considered more severe regardless of the final score. **Partial Mayo Index Score** Mucosal appearance at endoscopy is not included. ----------------------------------------- Remission: 0-1 [Remission defined as patient assessment of disease activity as perfect or very good (minimal symptoms).]^3^ Mild Disease: 2-4 Moderate Disease: 5-6 Severe Disease: 7-9 | Total |  |
| References | |  |
| 1. Rutgeerts P, Sandborn WJ, Feagan BG, Reinisch W, et al. Infliximab for induction and maintenance therapy for ulcerative colitis. N Engl J Med. 2005; 353 (23): 2462-2476. <https://www.ncbi.nlm.nih.gov/pubmed/16339095> | |  |

**Informed consent form**

מספר הבקשה בוועדת הלסינקי **:**

**0308-13-MMC**

אני החתום מטה:

|  | שם פרטי ומשפחה: |
| --- | --- |
|  | מספר תעודת זהות: |
| מיקוד: | כתובת: |

1. מצהיר/ה בזה כי אני מסכים/ה להשתתף בניסוי רפואי, כמפורט במסמך זה.
2. מצהיר/ה בזה כי איני משתתף בזמן חתימת מסמך זה, בניסוי רפואי אחר הכרוך בשימוש במוצר מחקר כלשהו, וכי אני מתחייב/ת לא להשתתף בכל ניסוי רפואי אחר הכרוך בשימוש במוצר מחקר במשך כל תקופת ניסוי זה.
3. מצהיר/ה בזה כי הוסבר לי על-ידי:

| שם החוקר/חוקר המשנה המסביר: |
| --- |

כי החוקר הראשי (שם הרופא):ד"ר תמנע נפתלי קיבל ממנהל המוסד הרפואי, בו ייערך הניסוי, אישור לביצוע הניסוי הרפואי בבני-אדם, כמשמעותו בתקנות בריאות העם (ניסויים רפואיים בבני-אדם) תשמ"א-1980 (להלן הניסוי הרפואי).

כי לחוקר הראשי או לחוקרי המשנה או למנהל המחלקה **יש זיקה** ^[[1]](#footnote-2)^ ליוזם הניסוי^[[2]](#footnote-3)^.

אם יש – פרט: החוקרת הראשית היא היוזמת

כי הניסוי הרפואי נערך בנושא:

מתן CANNABIS בעישון לחולי קוליטיס כיבית פעילה

כי אני חופשי/ה לבחור שלא להשתתף בניסוי הרפואי, וכי אני חופשי/ה להפסיק בכל עת את השתתפותי בניסוי, כל זאת מבלי לפגוע בזכותי לקבל את הטיפול המקובל.

כי במקרה של מילוי שאלון – אני רשאי/ת שלא לענות על כל השאלות שבשאלון או על חלק מהן.

כי מובטח לי שזהותי האישית תשמר סודית על-ידי כל העוסקים והמעורבים במחקר ולא תפורסם בכל פרסום, כולל בפרסומים מדעיים.

כי המוסד הרפואי פעל להסדרת כיסוי ביטוחי הולם של החוקרים, הרופאים והצוות הרפואי העוסקים בניסוי הקליני מפני תביעות שיוגשו ע"י משתתפים בניסוי הקליני ו/או תביעות צד ג' הקשורות עם הניסוי הקליני בין בתקופת ביצוע הניסוי ובין לאחריו. אין באמור כדי לפגוע בזכויותיי על פי כל דין.

כי במקרה הצורך, לפי המלצת החוקר הראשי, קיימת האפשרות שאמשיך לקבל את מוצר המחקר ללא תשלום גם לאחר סיום הניסוי הרפואי לתקופה של שלוש שנים, כאשר לא נמצא לי טיפול רפואי חליפי מתאים. זאת, בין היתר, בתנאי שהמוצר עדיין לא אושר לשימוש בהתוויה המבוקשת בניסוי במדינת ישראל, ולא ניתן לקבלו מקופת החולים/שירותי הבריאות בו/בהם אני מבוטח/ת.

ההחלטה לגבי המשך מתן מוצר המחקר נתונה בידי ועדת הלסינקי המוסדית, והכל כפוף לקיומו של תכנית טיפול ומעקב.

כי מובטחת לי נכונות לענות לשאלות שיועלו על-ידי וכן האפשרות להיוועץ בגורם נוסף (לדוגמא רופא-משפחה, בני משפחה וכו'), באשר לקבלת החלטה להשתתף בניסוי הרפואי ו/או להמשיך בו.

כי בניסויים רפואיים בהם משתתפות נשים בגיל הפוריות, במקרה של הריון במהלך הניסוי הרפואי, האישה תקבל ייעוץ (על-ידי החוקר) לגבי השפעות שיייתכנו על העובר ולגבי גורל ההריון, כולל האפשרות של הפסקת ההריון.

כי בכל בעיה הקשורה לניסוי הרפואי אוכל לפנות ל- ד"ר תמנע נפתלי

מספר טלפון/משיבון:09-7471054 , בכל שעות היממה.

1. הנני מצהיר/ה כי נמסר/ה לי מידע מפורט על הניסוי הרפואי, על פי הנושאים המפורטים להלן:
   1. **מטרות הניסוי;**

מטרת המחקר המוצע לבדוק יעילות של עישון Cannabis בחולי קוליטיס כיבית פעילה

- 1. **המספר בקירוב של המשתתפים בניסוי הרפואי;**

30 משתתפים

- 1. **התקופה הצפויה למשך ההשתתפות בניסוי;**

תקופת המחקר היא עשרה שבועות מתוכם בשמונת השבועות הראשונים המטופל מקבל סיגריות (קנאביס או פלסבו) ושבועיים נוספים ללא התכשיר. אם אין תגובה של המטופל לתכשיר אחרי ארבעה שבועות יש אפשרות להעביר אותו לזרוע פתוחה. מטופלים שמשתפר מצבם כתגובה לטיפול, והחוקרת הראשית חושבת שהם מתאימים, תשלח עבורם בקשה ליק"ר לרישיון קבוע לקנאביס רפואי והמעקב אחריהם ימשך שנה בכדי לבחון את ההשפעות ארוכת הטווח של קנאביס על המחלה ואת תופעות הלוואי ארוכות הטווח.

- 1. **שיטות;**

החולים יחולקו אקראית ל 2 קבוצות:

1. קבוצה אשר תקבל סיגריות מוכנות מצמח הקנביס לעישון פעמיים ביום

2. קבוצת אשר תקבל סיגריות דומות אך שמוצה מהן החומר הפעיל..

החולים יעברו בדיקה אנדוסקופית בתחילת ובסוף המחקר.

בנוסף ילקחו בדיקות דם בתחילת ובסוף המחקר להערכת פעילות וחומרת המחלה, יבדקו ספירת דם, CRP, וכימיה. כמו כן יבדקו הציטוקונים הבאים: 12-IL , 10-IL, TGF β לפני מתן תרופת המחקר ולאחר 8 שבועות של טיפול.

תלקח בדיקת צואה לקלפרוטקטין לפני המחקר ובסופו

- 1. **היתרונות הצפויים למשתתף או לאחרים, כתוצאה מהניסוי;**

אפשרות לקבל קנביס רפואי תוך מעקב והדרכה צמודים

- 1. **הסיכונים הידועים ו/או אי-הנוחות שניתן לחזותם למשתתף במחקר;**

צריכה של קנאביס עלולה לגרום לתופעות לוואי המתחלקות להשפעות פיזיולוגיות כמו: סחרחורת, דופק לא יציב (מואץ או איטי), חולשה, ירידה בלחץ דם וברמת הסוכר בדם, תיאבון מוגבר, אדמומיות בעיניים, כאבי ראש, כאבי בטן (כשנצרך בקפסולות), עייפות, קואורדינציה לקויה, חוסר יציבות ויובש באיברים ריריים כמו עיניים ופה. והשפעות קוגניטיביות כמו: איבוד זיכרונות בטווח קצר, פגיעה בקו מחשבה רציף ושינויים בתפיסת הזמן והמרחב. שימוש קבוע בכמויות גבוהות (יותר מ- 5 גרם ביום), עלול להוביל לפגיעה ביכולות הקוגניטיביות, אך השפעה זו נעלמת בהפסקת הצריכה.

תופעות אלו חולפות בדרך-כלל זמן קצר לאחר ההסתגלות לתכשיר. כשהקנאביס נצרך בבליעה או מציצה תופעות לוואי עלולות להמשך עד 72 שעות לאחר נטילת התכשיר.

תופעות לוואי הנובעות בדרך-כלל ממינון יתר, המחייבות התייחסות מיוחדת: עילפון, שינויים גדולים בלחץ הדם, בדופק, ברמות הסוכר בדם או בקצב הנשימה. מינון גבוה של החומר עשוי במקרים מסוימים, אצל אנשים בעלי נטייה מוקדמת, לגרום להתפרצות זמנית של מצבים פסיכוטיים, חרדה, הזיות או הלוצינציות.

אין לנהוג תחת השפעת קנביס, כמו כן קנביס אסור לנטילה בזמן הריון או בזמן נסיון להכנס להריון. במסגרת המחקר תתבקש/י לעבור שתי בדיקות אנדוסקופיות, בתחילת המחקר ובסופו, כדי להעריך האם הטיפול הביא לשיפור במצב הדלקת. בדיקות אלה מבוצעות לצורך המחקר ויתכן שללא המחקר לא היית צריך/ה לעבור אותן. עליך לדעת כי בדיקת הקולונוסקופיה כרוכה בסיכון קטן ביותר אך ממשי להווצרות של קרע במעי. במקרה הנדיר של הווצרות קרע במעי לאחר קולונוסקופיה יש לרוב צורך לעבור ניתוח לתיקון הקרע.

- 1. **נסיבות בהן עלולה השתתפותו בניסוי הרפואי להיפסק בהחלטת החוקר או היוזם;**

במידה ומצבך הרפואי יחמיר או שיהיה רושם שתרופת המחקר גורמת לנזק כלשהוא.

- 1. **לפי העניין, החוקר ימסור למשתתף מידע על תוצאות רפואיות אפשריות של החלטת המשתתף
     על הפסקת השתתפותו בניסוי הרפואי לפני סיומו;**

במידה ותחליט להפסיק את השתתפותך בניסוי תמשיך לקבל טיפול רפואי לפי מיטב שיקולנו והבנתנו וזכויותך או היחס אליך לא יפגעו כתוצאה מכך

- 1. **הסבר על טיפולים חלופיים, ועל יתרונותיהם וחסרונותיהם, באם ישנם כאלה, למשתתף;**

טיפולים במחלת הקוליטיס הכיבית כוללים תרופות מסוג 5-ASA (רפאסאל, פנטאזה , אסקול) סטרואידים, תיופורינים (אימוראן, פורינטול) ונוגדי TNF (רמיקייד, הומירה) תרופות אלה יעילות במרבית המקרים אך כרוכות בתופעות לוואי, בעיקר החלשה של מערכת החיסון

- 1. **מידע רלוונטי אחר (כפי שנמסר על-ידי יוזם הניסוי):**

כשמטופל נמצא מתאים למחקר נשלחת ליק"ר בקשה לרישיון לקנאביס רפואי במסגרת מחקר. בנוסף לבקשה מצורף טופס חתום ע"י המטופל- כתב ויתור סודיות שבו הוא חותם על כך שהוא לא ידרוש את המשך הטיפול באם הצוות הרפואי ו/ או משרד הבריאות יחליטו להפסיקו עבורו.

1. הנני מצהיר/ה בזה כי את הסכמתי הנ"ל נתתי מרצוני החופשי וכי הבינותי את כל האמור לעיל. כמו-כן, קיבלתי עותק של טופס הסכמה מדעת זה, נושא תאריך וחתום כדין.
2. עם חתימתי על טופס הסכמה זה, הנני מתיר ליוזם הניסוי הרפואי, לוועדת הלסינקי המוסדית, לגוף המבקר במוסד הרפואי ולמשרד הבריאות גישה ישירה לתיקי הרפואי, לשם אימות שיטות הניסוי הרפואי והנתונים הקליניים. גישה זו למידע הרפואי שלי תבוצע תוך שמירת סודיות, בהתאם לחוקים ולנהלים של שמירת סודיות.
3. במקרים שבהם הניסוי הרפואי כרוך במתן שירותים: ביצוע בדיקות רפואיות או באספקת אביזרים, תכשירים או משתלים, הנני מצהיר/ה בזה כי אני יודע/ת ומסכים/ה שהמידע על השתתפותי בניסוי הרפואי יועבר לרופא המטפל שלי בקופת החולים/שירותי הבריאות בה/בהם אני מבוטח/ת.

ידוע לי כי בקופת החולים/שירותי הבריאות לא ייעשה במידע זה כל שימוש, אלא לצורך טיפול ומעקב רפואיים בלבד.

| תאריך | חתימת המשתתף/ת בניסוי | שם המשתתף/ת בניסוי הרפואי |
| --- | --- | --- |
|  |  |  |

במקרה הצורך^[[3]](#footnote-4)^2

| תאריך | חתימת העד | מספר תעודת זהות | שם העד הבלתי תלוי |
| --- | --- | --- | --- |
|  |  |  |  |

הצהרת החוקר/חוקר המשנה:

ההסכמה הנ"ל נתקבלה על-ידי, וזאת לאחר שהסברתי למשתתף/ת בניסוי הרפואי כל האמור לעיל וכן וידאתי שכל הסבריי הובנו על-ידו/ידה.

| תאריך | חתימתו | שם החוקר/חוקר המשנה המסביר |
| --- | --- | --- |
|  |  |  |

1. קשר של העסקה בשכר, או קשר מסחרי או עסקי, או קשר משפחתי או אישי, וכל קשר אחר, לרבות קשר של כפיפות בעבודה, שיש בו כדי לעורר חשש לקיום ניגוד עניינים או תלות, ולמעט החזר הוצאות או תשלום עבור השתתפות בוועדות לפי נוהל זה. [↑](#footnote-ref-2)
2. אם החוקר הראשי הוא גם יוזם הניסוי, יש לציין זאת במפורש. [↑](#footnote-ref-3)
3. 2 במקרה שהמשתתף בניסוי, או נציגו החוקי, אינו מסוגל לקרוא את טופס ההסכמה מדעת, עד בלתי תלוי חייב להיות נוכח במשך ההסבר על מהות הניסוי הרפואי. לאחר שהמשתתף או נציגו החוקי הביע את הסכמתו בעל-פה להשתתפות בניסוי, העד יחתום על טופס ההסכמה, תוך ציון תאריך החתימה. [↑](#footnote-ref-4)
