# Supplementary material for: Cannabis is associated with clinical but not endoscopic remission in ulcerative colitis: A randomized controlled trial
Source: PLoS One. 2021 Feb 11;16(2):e0246871. doi: 10.1371/journal.pone.0246871 (PMC7877751; doi:10.1371/journal.pone.0246871)
Supplement: S2 File — (DOCX) [file pone.0246871.s003.docx]

מתן CANNABISבעישון לחולי קוליטיס כיבית פעילה

הצעה למחקר

פרוטוקול קנביס– גרסה 1 מתאריך 26/12/2013

דר תמנע נפתלי

המכון לגסטרואנטרולוגיה ומחלות כבד

בית חולים מאיר

כפר סבא

**רקע:** מחלת מעי דלקתית הינה מחלה נפוצה יחסית ושכיחותה עולה לאחרונה. הטיפולים למחלה כוללים תרופות שונות לדיכוי מערכת החיסון, החל מתכשיר ASA -5 , דרך קורטיקוסטרואידים וכלה בתרופות אימונומודולטוריות ותכשירים ביולוגיים נוגדי TNF. עם זאת לטיפול הקיים תופעות לוואי מרובות וחלק מן החולים אינם מגיבים למגוון התרופות הקיימות.

צמח הקנביס נמצא בשימוש המין האנושי מזה אלפי שנים, וכבר ברפואה הסינית העתיקה הוכרו תכונותיו כצמח מונע כאבים, מקל על שלשול ובעל אפקט נוגד דלקת. הצמח מכיל למעלה מ 60 מרכיבים שונים, שלא כולם בודדו או זוהו, עם זאת נראה שאת עיקר ההשפעה הפסיכו אקטיבית יש ליחס למרכיב teterhydrocannabinol 9Δ (THC ) ואילו ההשפעה נוגדת הדלקת מקורה כנראה ב cannabidiol (CBD)(^[[1]](#endnote-2)^).

בגוף קיימים גם קנבינואידים פנימיים כאשר העיקריים שבהם הינם Anandamide, ו-2 –arachidonoylglycerol (2-AG), מנגנוני הסינטזהשל שני אנדוקנבינואידים אלה נפרדים זה מזה, אך שניהם יכולים, לאחר שנוצרו, להתקשר לרצפטורים על גבי התא שיצר אותם , או לנדוד לתאים אחרים דרך הפלזמה, לעיתים על ידי התקשרות לחלבון נשא כגון אלבומין (^[[2]](#endnote-3)^.) רמות אנדוקנבינואידים ברקמות נשלטות בקפדנות על ידי מנגנון של re uptake והידרוליזה (^[[3]](#endnote-4)^). שני אנזימים אחראים בעיקר לפירוק האנדוקנבינואידים , הראשון הואamide hydrolaseFatty acid(FAAH)(^[[4]](#endnote-5)^), המצוי בכל הגוף אך ברמות גבוהות יותר במוח ובכבד. והשני monoacylglyceride lipase (MAGL)(^[[5]](#endnote-6)^), מצוי בטרמינלים של תאי עצב.

הן האנדוקנבינואידים והן הקנבינואידים הצמחיים פועלים על ידי קשירה לרצפטורים לקנבינואידים המצויים על תאים רבים בגוף. שני הרצפטורים העיקריים הינם CB1 ו 2CB, שני הרצפטורים שייכים למשפחה של G-proteincoupledreceptors. הרצפטור 1CB נמצא בעיקר על גבי טרמינלים של תאי עצב, הן במערכת העצבים המרכזית והן במערכת העצבים הפריפרית. (^[[6]](#endnote-7)^) אך גם בתאים של המערכת הוסקולרית, מערכת הרביה וחלק מן המערכת האנדוקרינית (^[[7]](#endnote-8)^). הרצפטור 2CB נמצא בעיקר על תאי מערכת הלימפה, בשכיחות גבוהה על גבי לימפוציטים מסוג B, פחות מזה על גבי נויטרופילים ומונוציטים, ובשכיחות עוד יותר נמוכה על גבי לימפוציטים מסוג T(^[[8]](#endnote-9)^).האנדוקנבינואידים מראים אפיניות שונה בקשירה לרצפטורים, Anandamide פועל כאגוניסט חלקי ומראה אפיניות גבוהה יותר ל 1CB ואילו 2-AG פועל כאגוניסט מלא ומראה אפיניותשוה לשניהם.

המערכת האנדוקנבינואידית נמצאת גם במערכת העיכול. רצפטורים לקנבינואידים אותרו במערכת העצבים האנטרית , כאשר רצפטורים מסוג 1CB אותרו במערכת העצבים המיאנטרית, הסובמוקוזלית, וכן בקצות תאי העצב המעצבבים את תאי השריר החלק האורכי והסיר קולרי (^[[9]](#endnote-10)^). רצפטורים מסוג 1CB נמצאו לא רק בתאי עצב במערכת העיכול אלא גם על גבי תאי אפיתל ותאי שריר חלק(^[[10]](#endnote-11)^). רצפטורים מסוג 2CB מבוטאים על גבי תאי פלסמה ומקרופגים משופעלים בלמינה פרופריה, על גבי תאי אפיתל וכן על תאי עצב בפלקסוסהמיאנטריוהסובמוקוזלי (^[[11]](#endnote-12)^(. רצפטורים אלה מתרבים (upregulated) במצבי דלקת כגון מודלים של מחלת מעי דלקתית(^[[12]](#endnote-13)^) או מחלת צליאק לא מטופלת (^[[13]](#endnote-14)^).

השפעות המערכת האנדוקנבינואידית על מערכת העיכול רבות ומגוונות ונוגעות כמעט לכל פאן של תפקוד מערכת זו(^[[14]](#endnote-15)^(. אגוניסטים לרצפטור 1CB מקטינים גרויכולינרגי ובכך מקטינים תנועתיות של מערכת העיכול. השפעה זו קיימת הן במעי בריא (^[[15]](#endnote-16)^) והן במעי מודלק ( ^[[16]](#endnote-17)^). קנבינואידים מקטינים שלשול וצבירת נוזלים במעי הנגרמים על ידי טוקסין הכולירע (^[[17]](#endnote-18)^). לקנבינואידים אפקט נוגד כאב משמעותי ביותר, והשפעתם גדולה הן בהקלת כאב סומטי והן בהקלת כאב ויסצרלי. הפעלה של רצפטורים 1CB ו 2CB מורידה את רגישות המעי הגס לכאב הנגרם מניפוח בלון במודל חולדה, במעי מודלק הפעלה של הרצפטורים 1CB ו 2CBמקטינה רגישות לכאב ( ^[[18]](#endnote-19)^), הפעלת הרצפטור 2CB מקטינה את הכאב הנגרם מברדיקינין (^[[19]](#endnote-20)^) . שימוש באנטגוניסט ספציפי ל 1CB , rimonabant, גרם להחמרת הכאב הנגרם מדלקת של המעי הגס (18 ), ממצא זה מרמז כי לקנבינואידים תפקיד מיוחד בהקטנת הכאב הנגרם מתהליכים דלקתיים באופן ספציפי.

בביופסיות מרקמת מעי של חולי IBD נמצא בטוי מוגבר של רצפטורים 1CB ו 2CB. במספר מודלים של מחלת מעי דלקתית במכרסמים נמצא כי אנדוקנבינואידים מקטינים את חומרת הדלקת במעי ( ^[[20]](#endnote-21)^, ^[[21]](#endnote-22)^) . במודל של עכברים עם קוליטיס שנגרמה על ידי sulfonic acid (TNBS)trinitrobenzene נמצא כי מתן אגוניסטים לרצפטור 2CB(JWH133, AM1241) הקל על הקוליטיס ואילו מתן אנטגוניסטים (AM630) החמיר אותה. בעכברים שהיו מחוסרי הרצפטור 2CB (-/-2CB ) לא הייתה לטיפול כל השפעה (^[[22]](#endnote-23)^ ) .

ניסויים שבדקו השפעת קנביס על רמות של ציטוקינים ופעילות תאי דלקת בבני אדם העלו תוצאות סותרות.מספר מחקרים שבהם ניתן קנביס בעישון לתקופה של מס שבועות לחולי איידס ולחולי טרשת נפוצה לא העלו הבדל ברמות הציטוקיניםTNF-α, IL-10, IL-12p40, and IL-12p70 , וכן לא היה הבדל בפרוליפרציה של תאי T או תאי פלסמה , מצד שני, במשתמשים שנטלו קנביס לזמן ממושך נמצא כי רמות תאי NK היו נמוכות יותר ותגובה של לימפוציטים למיטוגנים חלשה יותר. במקביל נצפתה גם ירידה ברמות 2-ILועליה ברמות 10-IL, כלומר הטיה של התגובה החיסונית מתגובה של 1-TH לתגובה של 2-TH ( ^[[23]](#endnote-24)^)

לקראת תכנון מחקר זה רואיינו 10 מטופלים הנוטלים קנביס רפואי עקב מחלת מעי דלקתית. כל המטופלים הצהירו כי השימוש בקנביס רפואי הביא לשיפור משמעותי בתסמיני המחלה ושיפור משמעותי באיכות חייהם. הערכה של מדד פעילות המחלה ( CDAI ) לפני ואחרי נטילת הקנביס הראתה ירידה בפעילות מממוצע של348 לממוצע של 108, כל המטופלים נזקקו לסטרואידים לפני תחילת השימוש בקנביס וכולם היו חופשיים מסטרואידים במהלך השימוש בו. 3 מטופלים עברו 5 ניתוחים לפני תחילת השימוש בקנביס ואף לא מטופל אחד נזקק לניתוח או אשפוז במהלך השימוש בקנביס. זמן ממוצע לשימוש היה 3.8 שנים (טווח 3 חודשים עד 9 שנים). לא נצפו כל תופעות לוואי משמעותיות. הממצאים הוצגו בכנס האיגוד הישראלי לגסטרואנטרולוגיה ומחלות כבד שהתקיים באילת ביוני 2009.

תופעות לוואי של שימוש רפואי במריחואנה הינן מועטות ורובן קלות. סקירה שפורסמה בקנדה סיכמה 23 מחקרים בהם ניתנה מריחואנה לחולים במחלות שונות בצורה מבוקרת ותחת מעקב. נסקרו 23 מחקרים מבוקרים ו 8 מחקרים תצפיתיים, אשר כללו נתונים מ 1932 מטופלים בקנביס רפואי. נמצא כי 96% מתופעות הלוואי שדווחו היו קלות , תופעת הלוואי השכיחה ביותר שדווחה על ידי 15% מהנחקרים (714/4779) היתה סחרחורת. תופעות לוואי קשות יותר נצפו בשכיחות זהה אצל מטופלים שקיבלו קנביס ואלה שקבלו אינבו. עם זאת זמן ההשתתפות במחקרים היה 12 חודשים לכל היותר ואין בידנו נתונים על ההשפעה של שימוש בקנביס לזמן ממושך יותר. המחקרים כולם לא כללו מתן קנביס בעישון אלא רק מתן תכשירי קנביס שונים דרך הפה. (^[[24]](#endnote-25)^) יש לציין כי קשה להעריך תופעות לוואי במתן קנביס שלא במסגרת טיפול רפואי שכן אנשים הצורכים קנביס מסיבות שאינן רפואיות נוטים גם לעשן טבק, לשתות אלכוהול ולצרוך סמים אחרים וקשה לבודד את השפעת הקנביס משאר ההשפעות (^[[25]](#endnote-26)^ ).

מכל האמור לעיל עולה כי המערכת האנדוקנבינואידית משתתפת ביצירה וקיום של תהליכים דלקתיים ועל כן יש הגיון רב בנסיון לרתום את המערכת לטיפול במצבים אלה. ישנם דיווחים אנקדוטליים רבים על הקלה בסימפטומים של מחלות מעי דלקתיות לאחר עישון מריחואנה (14) והרושם הוא כי יש לחומר אפקט מטיב על התהליך הדלקתי וכי תופעות הלוואי מועטות ביותר, עם זאת לא נערך מחקר מסודר בהשוואה עם אינבו בנושא זה.

מטרת המחקר המוצע לבדוק יעילות של עישוןCannabis בחולי קוליטיס כיבית לעומת אינבו.

חומר ושיטות:

אוכלוסית המחקר:

1.חולי קוליטיס כיבית, אשר המחלה קיימת אצלם מעל 3 חודשים, והוכחה בבדיקות המקובלות- אנדוסקופיה או הדמיה רנטגנית. בנוסף יכללו 10 חולים נוספים עם POUCHITIS (חולים לאחר כריתה שלמה של המעי הגס וחיבור לולאת מעי דק לפי הטבעת).

2. יוכנסו חולים בשלב פעיל של המחלה, אשר אינם מגיבים לטיפול בתכשירי 5 ASA או קורטיקוסטרואידים או אימונומודולטורים, או תרופות ביולוגיות. או שהפכו להיות תלויים בסטרואידים, או שאינם מסוגלים לקבל תרופות אלה עקב תופעות לוואי או תגובה אלרגית.

3. גיל המשתתפים במחקר 20 שנה או יותר.

4.דרגת פעילות המחלה תקבע לפי mayo score מעל 3 בחולי UC.

לא יוכנסו למחקר:

1. חולים עם הפרעות נפשיות ידועות,
2. חולים שלפי הערכת הרופא המטפל יש סיכון גבוה ליצירת התמכרות או שימוש לא ראוי בתכשיר
3. נשים בהריון או שמתכננות הריון.
4. חולים עם רגישות ידועה לתכשיר,
5. חולים שאינם מסוגלים לתת הסכמה מודעת,
6. חולים אשר נראה כי יזדקקו לניתוח בעתיד הקרוב עקב מחלתם.

במחקר יכללו סה"כ 40 חולי קוליטיס + 10 חולים עם POUCHITIS).

תרופה: החולים יחולקו אקראית ל 2 קבוצות:

1. קבוצה אשר תקבל סיגריות מוכנות מצמח הקנביס לעישון פעמיים ביום
2. קבוצת אשר תקבל סיגריות דומות אך שמוצה מהן החומר הפעיל..

משך המחקר יהיה 8 שבועות,

חולים אשר במהלך המחקר תחול החמרה במצבם: עליה של UCAI ביותר מ 2 נקודות יוצאו מן המחקר.

החולים יעברו בדיקה אנדוסקופית בתחילת ובסוף המחקר.

בנוסף ילקחו בדיקות דם בתחילת ובסוף המחקר להערכת פעילות וחומרת המחלה, יבדקו ספירת דם, CRP, וכימיה. כמו כן יבדקו הציטוקונים הבאים: 12-IL , 10-IL, TGF β לפני מתן תרופת המחקר ולאחר 8 שבועות של טיפול.

תלקח בדיקת צואה לקלפרוטקטין לפני המחקר ובסופו

תרופת המחקר וכן הפלצבו יסופקו מעמותת תיקון עולם, העמותה המספקת קנביס רפואי באישור משרד הבריאות. המחקר תואם ואושר הן על ידי העמותה והן על ידי דר יהודה ברוך, הרופא המוסמך מטעם משרד הבריאות למתן קנביס רפואי.

בנוסף- המשתתפים יתבקשו למלא שאלון תופעות לוואי ושאלון הערכה של התמכרות לתרופה. מהלך הבדיקות והביקורים במשך המחקר מפורט בטבלה שלהלן:

| **בקור** | **בקור I** | **בקור II** | **בקור III** | **בקור IV** |
| --- | --- | --- | --- | --- |
| **שבוע** | **0** | **2** | **8** | **10** |
| טופס הסכמה | V |  |  |  |
| אנמנזה ובדיקה גופנית | V | V | V | V |
| בדיקות דם: ספירה כימיהCRP ושקיעת דם | V | V | V | V |
| בדיקות ציטוקינים | V |  | V |  |
| בדיקת צואה לקלפרוטקטין | V |  | V |  |
| הערכת פעילות המחלה  CDAI/UCAI | V | V | V | V |
| שאלון איכות חיים | V |  | V |  |
| מתן תרופת המחקר | V | V |  |  |
| אנדוסקופיה (למי שמסכים) | V |  | V |  |
| שאלון תופעות לוואי |  |  | V |  |

**טופס מעקב אחרי משתתפים במחקר : מחלות מעי דלקתיות וקנביס**

**תאריך: ______ רופא מפנה: _________**

**בקור I:**

| **שם:** |  | **משפחה:** |  |
| --- | --- | --- | --- |
| **ת.ז.:** |  | **שנת לידה:** | **מין : ז/נ** |
|  |  | **טלפון:** |  |
| **מחלות רקע:** |  |  |  |
| **תרופות קבועות:** |  |  |  |
| **עישון: מעולם לא** | **בעבר, תאריך הפסקה** |  | **בהווה:** |
| **IBD במשפחה** | **כן/לא** | **איזו קרבה:** |  |
|  |  | **איזו מחלה:** |  |
| **היסטוריה של IBD:** |  | **UC** | **Indeterminate** |
| **תאריך אבחנה I:** |  |  |  |
| **אובחן עי:** | **אנדוסקופיה** | **צ מעי דק** | **CT** |
|  | **ביופסיה** | **אחר:** |  |
| **ניתוחים: כן/לא** | **תאריך:** | **איזה ניתוח:** |  |
| **טיפול תרופתי בעבר:** | **(לפני יותר מ 1 חודש)** | |  |
| **ASA-5** | **בן/לא** | **סטרואידים** | **כן/לא** |
| **אנטיביוטיקה** | **כן/לא** | **אימורן/6MP** | **כן/לא** |
| **נוגדי TNF** | **כן/לא**  **תאריך ערוי אחרון:** | **מטוטרקסט** | **כן/לא** |
| **קטע מעי מעורב:** | **פרוקטיטיס/ מעי גס שמאלי בלבד / מעי גס שמאלי +רוחבי/ כל המעי הגס/ אילאום טרמינלי/ אילאום/ גגונום/ תריסריון/ קיבה/ ושט** | | |
|  |  | | |
|  |  | | |
| **מחלה נוכחית:** | **מולא /UCAI** |  |  |
| **אנדוסקופיה: (במידה ובוצעה)** | |  | |
| **דירוג חומרת התמונה האנדוסקופית:** | |  | |
| **נלקחו ביופסיות:** | **מעי גס כן/לא** | **אילאום טרמינלי** | **כן/לא** |
| **טיפול תרופתי נוכחי:**  **(חודש אחרון)** | **אנטיביוטיקה: כן/לא** | **סוג אנטיביוטיקה:** |  |
|  |  | **תאריך התחלה** |  |
|  |  | **תאריך סיום:** |  |
|  | **טיפול מקומי: פרט** |  |  |
|  | **ASA-5 כן/לא** | **תאריך התחלה** |  |
|  |  | **תאריך סיום:** |  |
|  | **סטרואידים כן/לא** | **תאריך התחלה** |  |
|  |  | **תאריך סיום:** |  |
|  | **אימורן/6MP** | **תאריך התחלה** |  |
|  |  | **תאריך סיום:** |  |
|  | **נוגדי TNF** | **תאריך התחלה** |  |
|  |  | **תאריך סיום:** |  |
| **מילא UCAI** |  | **בדיקות דם** |  |
| **מילא שאלון איכות חיים** | | **אנדוסקופיה** |  |
|  |  |  |  |

**בקור II (שבוע 2) תאריך: ________**

| אנמנזה ובדיקה גופנית |  | | | | | |
| --- | --- | --- | --- | --- | --- | --- |
| תרופות שנוטל: | תרופה | מינון | דרך מתן | אינדיקציה | התחלה | סיום |
|  |  |  |  |  |  |  |
| שינוי בטיפול התרופתי: |  |  |  |  |  |  |
| בדיקות דם: | ספירה כימיה CRP ושקיעת דם | | | | | |
| הערכת פעילות המחלה  /UCAI |  | | | | | |
| מתן תרופת המחקר |  | | | | | |
| מועד לביקור הבא: |  | | | | | |

**בקור III (שבוע 8) תאריך: ________**

| אנמנזה ובדיקה גופנית |  | | | | | |
| --- | --- | --- | --- | --- | --- | --- |
| תרופות שנוטל: | תרופה | מינון | דרך מתן | אינדיקציה | התחלה | סיום |
|  |  |  |  |  |  |  |
| שינוי בטיפול התרופתי: |  |  |  |  |  |  |
| בדיקות דם: | ספירה כימיה CRP ושקיעת דם, | | | | | |
| בדיקת ציטוקינים |  | | | | | |
| הערכת פעילות המחלה  /UCAI |  | | | | | |
| אנדוסקופיה (למי שמסכים) |  | | | | | |
| מועד לביקור הבא: |  | | | | | |

**בקור IV (שבוע 10) תאריך: ________**

| אנמנזה ובדיקה גופנית |  | | | | | |
| --- | --- | --- | --- | --- | --- | --- |
| תרופות שנוטל: | תרופה | מינון | דרך מתן | אינדיקציה | התחלה | סיום |
|  |  |  |  |  |  |  |
| שינוי בטיפול התרופתי: |  |  |  |  |  |  |
| בדיקות דם: | ספירה כימיה CRP ושקיעת דם | | | | | |
| הערכת פעילות המחלה  /UCAI |  | | | | | |

Disease activity index for UC

| Symptom | **Details** | **Points** |
| --- | --- | --- |
| No of bowel movements | 0-2 | 0 |
|  | 3-4 | 1 |
|  | 6-5 | 2 |
|  | 9-7 | 3 |
|  | 10 | 4 |
| Nocturnal diarrhea | No | 0 |
|  | yes | 1 |
| Blood in stools (% of Time) | None | 0 |
|  | Less than 50% | 1 |
|  | More than 50% | 2 |
|  | 100% | 3 |
| Incontinence | no | 0 |
|  | yes | 1 |
| Abdominal pain | none | 0 |
|  | mild | 1 |
|  | moderate | 2 |
|  | severe | 3 |
| General well being | Perfect | 0 |
|  | Very good | 1 |
|  | Good | 2 |
|  | Average | 3 |
|  | Poor | 4 |
|  | terrible | 5 |
| Abdominal tenderness | None | 0 |
|  | Mild and localized | 1 |
|  | Moderate and diffuse | 2 |
|  | Severe or rebound | 3 |

References:

1. Fernando Rodriguez de Fonseca, Ignacio Del Arco ,Francisco Javier Bermudez-Silva, Ainhoa Bilbao,AndreaCippitelli MiguelNavaro. The Endocannabinoid System: Physiologyand Pharmacology. Alcohol & Alcoholism 2005 . **40**, 2–14. [↑](#endnote-ref-2)
2. Piomelli, D. The molecular logic of endocannabinoid signalling. Nature Reviews Neuroscience **2003 4**, 873–884. [↑](#endnote-ref-3)
3. Beltramo, M., Stella, N., Calignano, A., Lin, S. Y., Makriyannis, A. andPiomelli, D. Functional role of high-affinity anandamidetransport, as revealed by selective inhibition. Science 1997**277**,1094–1097. [↑](#endnote-ref-4)
4. Cravatt, B. F., Giang, D. K., Mayfield, S. P., Boger, D. L., Lerner, R. A.and Gilula, N. B. Molecular characterization of an enzymethat degrades neuromodulatoryfatty-acid amides. Nature 1996 **384**,83–87. [↑](#endnote-ref-5)
5. Dinh, T. P., Carpenter, D., Leslie, F. M., Freund, T. F., Katona, I.,Sensi, S. L., athuria, S. and Piomelli, D. Brainmonoglyceride lipase participating in endocannabinoid inactivation.Proceedings of the National Academy of Sciences of the United States of America 2002 **99**, 10819–10824. [↑](#endnote-ref-6)
6. Devane, W. A., Dysarz, F. A. 3rd, Johnson, M., Melvin, L. S. and Howlett, A. C. Determination and characterization of a cannabinoid receptor in rat brain. Molecular Pharmacology 1988 **34**, 605–613. [↑](#endnote-ref-7)
7. Howlett, A. C., Bidaut-Russell, M., Devane, W. A., Melvin, L. S., Johnson, M. R. and Herkenham, M. The cannabinoid receptor: biochemical, anatomical and behavioral characterization. Trends in Neurosciences 1990 **13**, 420–423. [↑](#endnote-ref-8)
8. Galiègue, S., Mary, S., Marchand, J., Dussossoy, D., Carrière, D., Carayon, P., Bouaboula, M., Shire, D., Le Fur, G. and Casellas, PExpression of central and peripheral cannabinoid receptors in human immune tissues and leukocyte subpopulations. European Journal of Biochemistry 1995 **232**, 54–61. [↑](#endnote-ref-9)
9. Duncan M, Davison JS, Sharkey KA. Endocannabinoids and their receptors in the enteric nervous system. Aliment PharmacolTher 2005;**22**:667–83. [↑](#endnote-ref-10)
10. Wright K, Rooney N, Feeney M, et al. Differential expression of cannabinoid receptors in the human colon: cannabinoids promote epithelial wound healing. Gastroenterology 2005;**129**:437–53. [↑](#endnote-ref-11)
11. Wright KL, Duncan M, Sharkey KA. Cannabinoid CB(2) receptors in thegastrointestinal tract: a regulatory system in states ofinflammation. Br J Pharmacol 2008;153:263–70. [↑](#endnote-ref-12)
12. Wright KL, Duncan M, Sharkey KA. Cannabinoid CB(2) receptors in the gastrointestinal tract: a regulatory system in states ofinflammation. Br J Pharmacol 2008;**153**:263–70. [↑](#endnote-ref-13)
13. Wright K, Rooney N, Feeney M, et al. Differential expression of cannabinoid receptors in the human colon: cannabinoids promote epithelial wound healing. Gastroenterology 2005;**129**:437–53. [↑](#endnote-ref-14)
14. A AIzzo and M Camilleri Emerging role of cannabinoids in gastrointestinal and liver diseases: basic and clinical aspects *Gut* 2008;57;1140-1155; [↑](#endnote-ref-15)
15. Esfandyari T, Camilleri M, Busciglio I, et al. Effects of acannabinoid receptor agonist on colonic motor and sensoryfunctions in humans: a randomized, placebo-controlled study. .Am J PhysiolGastrointest Liver Physiol 2007;293:G137–45. [↑](#endnote-ref-16)
16. Mathison R, Ho W, Pittman QJ, et al. Effects of cannabinoidreceptor-2 activation on accelerated gastrointestinal transit inlipopolysaccharide-treated rats. Br J Pharmacol

    2004;142:1247–54 [↑](#endnote-ref-17)
17. Izzo AA, Capasso F, Costagliola A, Bisogno T, Marsicano G, LigrestiA, Matias I, Capasso R, Pinto L, Borrelli F, Cecio A, Lutz B, MascoloN, DiMarzo V. An endogenous cannabinoid tone attenuates choleratoxin-induced fluid accumulation in mice. Gastroenterology2003;125:765–774. [↑](#endnote-ref-18)
18. Sanson M, Bueno L, Fioramonti J. Involvement of cannabinoidreceptors in inflammatory hypersensitivity to colonic distension inrats. NeurogastroenterolMotil 2006;18:949–56. [↑](#endnote-ref-19)
19. Hillsley K, McCaul C, Aerssens J, et al. Activation of thecannabinoids 2 (CB2) receptor inhibits murine mesenteric afferentnerve activity. NeurogastroenterolMotil 19:769–77. [↑](#endnote-ref-20)
20. Massa F, Marsicano G, Hermann H, et al. The endogenouscannabinoid system protects against colonic inflammation. J ClinInvest 2004;113:1202–9. [↑](#endnote-ref-21)
21. Kimball ES, Schneider CR, Wallace NH, et al. Agonists ofcannabinoid receptor 1 and 2 inhibit experimental colitis inducedby oil of mustard and by dextran sulfate sodium. Am J PhysiolGastrointest Liver Physiol 2006;291:G364–71. [↑](#endnote-ref-22)
22. Martin A. Storr, Catherine M. Keenan, Hong Zhang, Kamala D Patel, Alexandros Makriyannis, and Keith A. Sharkey, Activation of the Cannabinoid 2 Receptor (CB2) Protects Against Experimental Colitis Inflamm Bowel Dis 2009. [↑](#endnote-ref-23)
23. Dai Lu, V. Kiran Vemuri, Richard I. Duclos, Jr. and Alexandros Makriyannis The Cannabinergic System as a Target for Anti-inflammatory Therapies. Current Topics in Medicinal Chemistry, 2006, Vol. 6, No. 13 [↑](#endnote-ref-24)
24. Wang T, Collet JP, Shapiro S, et al. Adverse effects of medical cannabinoids: a systematic review. CMAJ 2008;178:1669-78. [↑](#endnote-ref-25)
25. Degenhardt L, Hall W D, The adverse effects of cannabinoids: implications for use of medical marijuana CMAJ 2008; 178:1685-1686 [↑](#endnote-ref-26)
